# Implicit Bias in Health Professionals: A Scoping Review

**DOI:** 10.3390/ijerph23070840

**Published:** 2026-06-26

**Authors:** Kelly Chacon-Acevedo, Ana María Castillo, John Alexander Castro-Muñoz, Yonatan Ferney Rojas, Andrea Bermudez-Rodriguez, Ana María Rojas-Gómez

**Affiliations:** 1 Center for Evidence Evaluation, Research for Health Decisions (CEIDS), Translational Research Group, Keralty Global Institute for Health Care Excellence (IGEC-K), Bogotá 110111, Colombia; krchacon@keralty.com; 2 Keralty Global Institute for Health Care Excellence (IGEC-K), Bogotá 110111, Colombia; anacastillog@gmail.com; 3 Family Medicine Postgraduate Programme, Fundación Universitaria Sanitas, Bogotá 110131, Colombia; 4 Department of Psychology, Social Sciences and Education Fundación Universitaria Sanitas, Bogotá 110131, Colombia; johacastro@unisanitas.edu.co (J.A.C.-M.); yfrojas@unisanitas.edu.co (Y.F.R.); 5 Models and Programs Unit, Keralty Global Institute for Health Care Excellence (IGEC-K), Bogotá 110111, Colombia; apbermudez@keralty.com

**Keywords:** implicit bias, health professionals, scoping review, Implicit Association Test, measurement, health equity, evidence gap map, PRISMA-ScR

## Abstract

**Highlights:**

**Public health relevance—How does this work relate to a public health issue?**

- Implicit bias in healthcare professionals contributes to inequities in clinical communication, diagnostic pathways, and treatment decisions, which can accumulate into measurable population-level disparities.
- This scoping review maps how implicit bias is being assessed across professions, settings, and bias targets, helping to identify where evidence exists (and where it is missing) to address inequities in health systems.

**Public health significance—Why is this work of significance to public health?**

- The review highlights major methodological heterogeneity (tools, administration, scoring, and reporting) that limits comparability and weakens the evidence base needed to design and scale effective equity interventions.
- By synthesizing constructs, targets, and contexts of bias measurement, the study supports surveillance and evaluation strategies that are essential for monitoring progress toward equitable care at the system level.

**Public health implications—What are the key implications or messages for practitioners, policy makers and/or researchers in public health?**

- Practitioners and educators should prioritize standardized, evidence-based administration and reporting of implicit measures and integrate bias mitigation as a longitudinal competency rather than a single-session training activity.
- Researchers and policy makers should invest in developing and validating measurement approaches that better capture truly automatic, context-robust responses and link bias metrics to patient-centered and equity-relevant outcomes.

**Abstract:**

Implicit bias, automatic attitudes or stereotypes outside conscious awareness, may influence clinicians’ communication, diagnosis, and treatment decisions, contributing to inequities in care. We conducted a scoping review to map measurement strategies used to assess implicit bias among health professionals and students in healthcare and training settings. Using Joanna Briggs Institute guidance and PRISMA-ScR, we searched PubMed, Embase, BVS, Google Scholar, and institutional repositories for studies to November 2025; two reviewers independently screened and charted data (protocol was developed a priori but submitted internal in organization, and then uploaded in OSF. Of 1864 records, 93 studies from 28 countries were included. We identified 57 bias domains, most often race/ethnicity, weight, and sexual orientation. Across studies, 42 unique instruments were reported; the Implicit Association Test was most common, while psychometric validation and administration details were frequently limited, constraining comparability and interpretation. Evidence gap mapping showed concentration in academic and hospital settings, with fewer studies in primary care or community contexts and limited attention to age, disability, and intersectionality-related biases. The evidence base is growing but fragmented; future work should prioritize standardized administration and reporting, stronger validation, and tools that better capture automatic responding across diverse identities and care settings to support education and equity-oriented interventions.

## 1. Introduction

The Implicit bias refers to automatic associations or evaluations toward social groups that can be activated without conscious awareness and that may influence perception, judgment, and behavior [1,2,3,4]. These associations are shaped by social structures, cultural norms, and historical contexts, and may persist even when individuals explicitly endorse egalitarian values [5,6,7,8]. Within cognitive and social psychology, implicit bias has been conceptualized as a form of mental representation, often involving stereotypes or prejudicial associations, that operates rapidly and without deliberate intent [4,9,10,11].

Implicit and explicit biases are related but conceptually distinct constructs. Explicit bias reflects attitudes and beliefs that individuals can consciously recognize and report, typically assessed through self-report questionnaires. In contrast, implicit bias captures more automatic, often non-conscious associations that may not align with stated beliefs. Because explicit measures are susceptible to social desirability and self-presentation, particularly in professional and socially regulated environments, implicit measures have been increasingly used as a complementary approach to examine attitudes that may influence decision-making under conditions of time pressure, cognitive load, or uncertainty [3,12,13].

The automated nature of implicit bias means that individuals who hold such associations may not critically reflect on them or even recognize their presence [14]. As a result, implicit biases can be difficult to access, acknowledge, and measure, posing a methodological challenge for both research and applied settings [14,15,16]. This challenge is especially relevant in healthcare, where clinical interactions are embedded within institutional cultures and broader social contexts that may reinforce existing power structures and inequities [7,17,18].

In healthcare settings, implicit bias has been associated with differences in communication, clinical judgment, and treatment decisions, with potential downstream effects on patient experiences and outcomes [19,20,21,22,23]. These effects are particularly concerning given evidence that implicit biases may contribute to the persistence and reinforcement of health disparities, disproportionately affecting marginalized populations [22,23,24]. For example, the Medical Student Cognitive Habits and Growth Evaluation Study (CHANGES) found that a substantial proportion of U.S. medical students exhibited both implicit and explicit weight bias, with levels of implicit weight bias comparable to those observed for racial bias in the same cohort [25,26].

These findings carry significant public health implications. Racial and ethnic bias in healthcare professionals has been linked to undertreated pain in Black patients, lower rates of cardiac procedure referrals for minority patients, and persistent disparities in maternal mortality [22,27]. Weight bias is associated with patient avoidance of care, reduced screening uptake, and shorter clinical consultations, all of which can delay diagnosis and worsen chronic disease outcomes [26]. Gender bias has been associated with underdiagnosis of cardiovascular disease in women and differential treatment of pain by sex. Collectively, these forms of bias do not operate in isolation: they compound across encounters and systems, accumulating into measurable population-level disparities. Mapping how these biases are measured is therefore not merely a methodological exercise; it is a prerequisite for designing, implementing, and evaluating the equity-oriented interventions that health systems urgently need.

Considerable effort has been devoted to measuring and mitigating implicit bias in healthcare; however, evidence of sustained effectiveness remains limited, as reflected in the continued presence of bias and its consequences [7,8,26]. One proposed explanation for these mixed results relates to challenges in measurement, including variability in how implicit bias is operationalized, assessed, and interpreted [28,29,30,31]. Measurement tools may differ in their sensitivity to conscious control, contextual influences, and task familiarity, which can affect the validity and comparability of findings across studies.

A wide range of methods has been used to operationalize and measure implicit bias. Reaction-time paradigms, most notably the Implicit Association Test (IAT), are among the most widely used approaches in health-professions research, alongside variants such as the Brief IAT and Single-Category IAT, as well as other paradigms including the Go/No-Go Association Task, evaluative priming, and the Affect Misattribution Procedure [32,33,34,35,36,37,38]. IAT results are commonly summarized using the D-score, a standardized metric derived from differences in response latencies between congruent and incongruent pairing blocks, with larger absolute values indicating stronger automatic associations [15]. Despite their widespread use, studies vary substantially in how these tools are implemented, scored, and reported, including inconsistent reporting of psychometric properties such as reliability and validity.

Importantly, implicit bias in healthcare can target multiple domains (or bias targets), including race/ethnicity, gender, weight, disability, age, mental health status, socioeconomic position, and sexual orientation or gender identity [27,30]. These domains may operate differently across clinical contexts and professional roles, and measurement tools are often developed to assess specific targets or settings [30]. As a result, the existing evidence base may overrepresent certain forms of bias while leaving others underexplored [31].

Given the breadth of constructs (e.g., attitudes, stereotypes, cognition), bias domains, and healthcare settings in which implicit bias has been studied, a scoping review is well suited to map the literature, clarify how implicit bias is operationalized, and identify gaps in measurement and reporting. Therefore, this scoping review aimed to: (1) catalog the instruments and approaches used to assess implicit bias among health professionals and healthcare students; (2) describe which constructs, bias domains, and settings these tools address; and (3) summarize how studies report scoring and psychometric properties of these measures.

## 2. Materials and Methods

### 2.1. Protocol and Registration

We conducted a scoping review to systematically map the existing literature on implicit bias among healthcare professionals. This review followed the methodological guidance outlined by the Joanna Briggs Institute (JBI) for scoping reviews [39] and adhered to the PRISMA Extension for Scoping Reviews (PRISMA-ScR) [40] checklist. The review protocol was developed a priori but submitted internally in organization with the project (See Institutional Review Board Statement), and then uploaded in OSF (available at: https://osf.io/yfbjd/overview?view_only=255bffd736cb4402b9e75dd771ca6862) (accessed on 14 March 2026).

### 2.2. Eligibility Criteria

We defined eligibility criteria using the Population–Concept–Context (PCC) framework:

- Population: Health professionals including physicians, nurses, allied health workers, and healthcare students.
- Concept: Implicit bias (e.g., unconscious stereotypes, attitudes, or perceptions) measured through validated or adapted tools.
- Context: Healthcare-related settings, including clinical practice, educational environments, and training programs.
- Types of sources: We included primary research studies (quantitative, qualitative, and mixed methods), as well as instrument development and validation studies. Editorials, commentaries, and conference abstracts were excluded unless they contained original data

Excluded references: Studies without an instrument, studies measuring only explicit biases, or those that did not address the research question.

### 2.3. Information Sources and Search

We conducted a systematic literature search across the following electronic databases: PubMed, Embase and BVS. Grey literature was retrieved through Google Scholar and institutional repositories. The Google Scholar search used free-text terms without restriction by journal type, language, or discipline, allowing capture of publications beyond biomedical databases (first 200 results per query screened). The final search was conducted in November 2025. No lower date limit was applied; the search retrieved records from database inception. The complete search strategies for each database are provided in Supplementary File S1, Tables S1–S4.

### 2.4. Selection of Sources of Evidence

**Figure 1:**
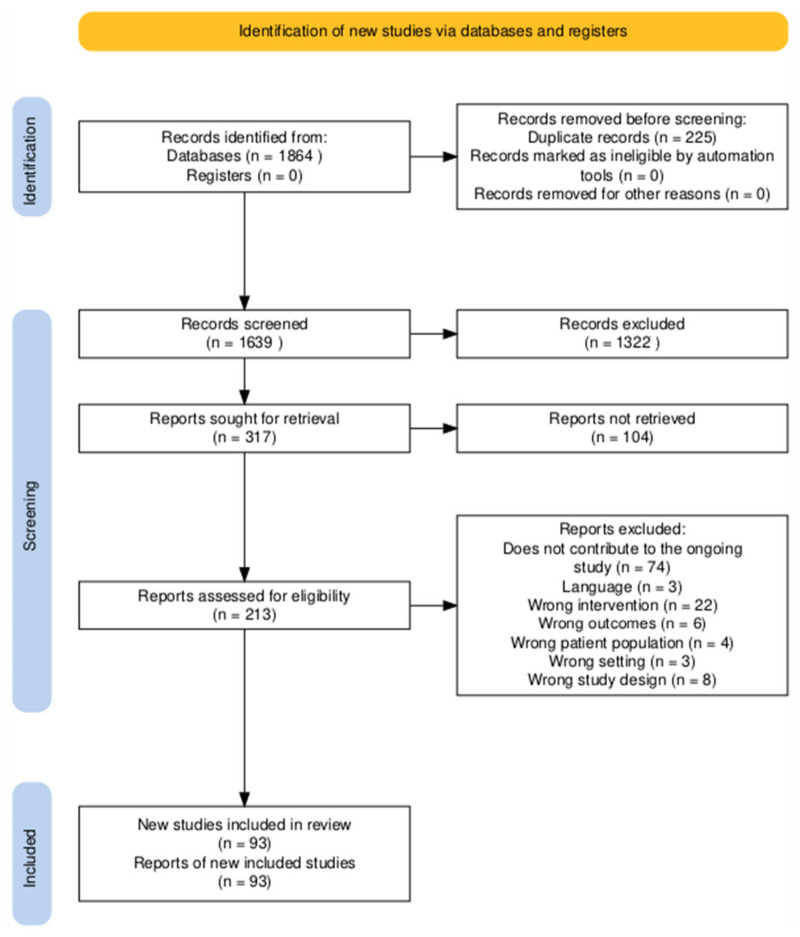
Flowchart PRISMA 2020 flow diagram for this scoping review (database and register searches only). Source: Page MJ et al. [41]. This work is licensed under CC BY 4.0. To view a copy of this license, visit https://creativecommons.org/licenses/by/4.0/ (accessed on 10 January 2026).

All identified references were imported into Rayyan (Rayyan Systems Inc., Cambridge, MA, USA) for deduplication and screening (AMRG, KCA). Two blinded reviewers, working in pairs (AMRG, KCA, AMC, JACM, YFR, ABR), independently screened titles and abstracts, followed by full-text review. Any disagreements were resolved by discussion or third-party adjudication (AMRG, KCA). Reasons for exclusion at the full-text stage were documented. The study selection process is illustrated using a PRISMA-ScR flow diagram (Figure 1).

### 2.5. Data Charting Process

A standardized data charting form was developed and piloted collaboratively by the review team. Data extraction was conducted by two reviewers independently and included the following variables: Reference (author, year, country); Study population and setting; Type of bias assessed; Instrument used, Methodological characteristics and constructs evaluated; Key findings related to implicit bias; Instrument subscales, operational performance, and psychometric properties.

For studies using the IAT, we extracted the reported D-score, a standardized scoring metric derived from differences in response latencies between congruent and incongruent pairing blocks. In general, the sign and magnitude of the D-score indicate the relative strength of automatic associations (e.g., stronger association of one social group with “good” versus “bad”), with larger absolute values reflecting stronger associations.

### 2.6. Synthesis of Results

We conducted a descriptive and thematic synthesis of the extracted data. Quantitative findings were summarized using frequencies and tabular presentations. Thematic categories were generated inductively to describe patterns in instruments used, constructs evaluated, and populations studied. Results are presented narratively and supported by summary tables and illustrative quotes where applicable. Evidence maps were generated using R version 4.4.2.

Language editing support was provided by ChatGPT (GPT-4, OpenAI, San Francisco, CA, USA, 2024) and Claude Sonnet 4.5 (Anthropic, San Francisco, CA, USA, 2025) to improve grammar, clarity, and fluency. The final content was reviewed and approved by the authors.

### 2.7. Critical Appraisal of Sources of Evidence

Consistent with scoping review methodology and the objectives of mapping the existing evidence, a formal critical appraisal of the methodological quality of included studies was not conducted.

## 3. Results

### 3.1. Selection of Sources

A total of 1864 records were retrieved through database searching. After removal of duplicates (*n* = 225), 1639 titles and abstracts were screened. Of these, 317 full-text reports were sought for retrieval, and 104 could not be accessed; for each inaccessible record, the team made two retrieval attempts: (1) an inter-library loan request through the institutional library, and (2) a direct email to the corresponding author. Records that remained unavailable after both attempts were excluded. A total of 213 full-text reports were assessed for eligibility, of which 120 were excluded for reasons such as irrelevance to the study topic (*n* = 74), wrong intervention or outcomes (*n* = 28), or ineligible populations or settings (*n* = 15). An overview of these excluded publications is presented in the Supplementary File, Table S5. Finally, 93 references were included in the final review. The full PRISMA 2020 flow diagram is shown in Figure 1.

### 3.2. Characteristics of Sources of Evidence

The included studies were published between 2007 and 2025. Most studies were conducted in the United States (*n* = 52; 55.9%), followed by Canada (*n* = 9; 9.7%) and the United Kingdom (*n* = 6; 6.5%); the remaining studies were distributed across other countries (Table 1).

Populations studied included physicians, nurses, therapists, residents, students, and a wide range of healthcare professionals working in emergency, primary care, hospital-based, or academic settings. A detailed list of included studies and their key characteristics is provided in Supplementary File S1, Table S6.

A wide diversity of biases were explored. Among the 57 types of bias or target domains identified, the most common included (Table 1):

- Race/ethnicity-related bias;
- Bias toward patients with specific diseases (e.g., mental illness, HIV, chronic pain);
- Weight-related stigma;
- Sexual orientation and gender identity;
- Disability-related bias;
- Age.

The most frequently used instrument was the Implicit Association Test (IAT) (Table 2), reported in over one-third of studies. In total, 42 unique instruments were identified across the included sources, including the Health Care Provider HIV/AIDS Stigma Scale (HPASS), the Attitudes Toward Obese Persons Scale (ATOP), the Genderism and Transphobia Scale (GTS), and the Mental Illness Clinician’s Attitudes Scale (MICA-4).

The most frequently assessed by the identified instruments were race/ethnicity and gender followed by weight. The IAT, and its variants (BIAT, SC-IAT) were used in the largest proportion of included studies (*n* = 40; approximately 43%), making them the dominant measurement approach by a considerable margin. The next most frequently identified instruments were clinical vignettes (*n* = 8), followed by self-report Likert-type scales including the Beliefs About Obese Persons Scale (BAOP; *n* = 3), the Health Care Provider HIV/AIDS Stigma Scale (HPASS; *n* = 3), the Opening Minds Scale for Health Care Providers (OMS-HC; *n* = 3), the Attitudes Toward Obese Persons Scale (ATOP; *n* = 2), and the Antifat Attitudes questionnaire (AFA; *n* = 2). The Genderism and Transphobia Scale (GTS; *n* = 1), and the Mental Illness Clinician’s Attitudes Scale (MICA-4; *n* = 1), were each identified in a single study. Beyond the IAT family, two additional performance-based implicit measures were identified: the Diabetes Provider Implicit Bias tool (D-PIB; *n* = 1) and the Implicit Relational Assessment Procedure (IRAP; *n* = 1), the latter reported in a narrative review of measurement approaches across career stages in medical education [88].

A full summary of instrument frequency and psychometric reporting across all 93 studies is presented in Table 3; detailed administration and scoring information for performance-based implicit measures is provided in Table 2.

Other measures identified in this review were Likert-type scales assessing attitudes and beliefs, with only a small subset of studies conducting formal criterion validity analyses in comparison to the IAT. Overall, psychometric information, including reliability and construct validity, was rarely described in the included studies.

**Figure 2:**
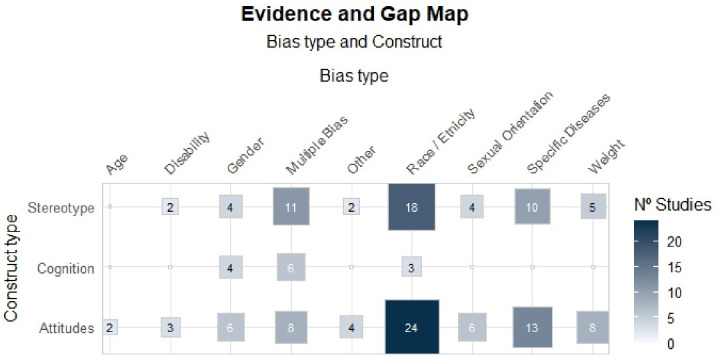
Evidence gap map by construct and bias type. Distribution of studies assessing implicit bias among healthcare professionals, organized by construct (attitudes, stereotypes, cognition) and bias target (race/ethnicity, weight, gender, etc.). Darker colors indicate a higher number of studies; blank cells indicate no studies identified.

**Figure 3:**
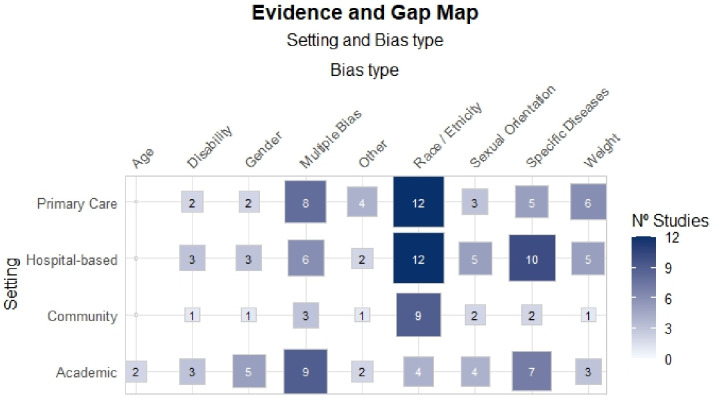
Evidence gap map by setting and bias type. Distribution of studies assessing implicit bias across different healthcare settings (academic, hospital, primary care, community) and bias target. Color intensity corresponds to the number of studies per cell.

Figure 2 maps bias constructs against bias types, showing that attitudes related to race/ethnicity dominate the literature (*n* = 24), followed by attitudes toward specific diseases (*n* = 13) and weight (*n* = 8). Race/ethnicity was the most frequently assessed bias target across constructs, particularly in studies examining attitudes, whereas disability and age were rarely explored (≤4 studies each), leaving notable evidence gaps. Figure 3 maps care settings against bias types, with hospital-based (*n* = 12 for race/ethnicity; *n* = 10 for specific diseases) and academic settings (*n* = 9 for multiple bias; *n* = 7 for specific diseases) most frequently studied, while primary care and community settings had consistently fewer than six studies per bias type.

## 4. Discussion

This review makes several contributions that distinguish it from prior work. Three prior reviews have examined implicit bias measurement in healthcare settings: FitzGerald and Hurst (2017) [30] conducted a systematic review identifying 15 studies primarily using the IAT; Maina et al. (2018) [8] focused specifically on racial/ethnic IAT studies over a decade; and Meidert et al. (2023) [135] conducted a scoping review including 38 studies. The present review extends this evidence base in four important respects. First, we identified 42 unique instruments across 93 studies, providing the most comprehensive instrument mapping to date. Second, we included the BVS/LILACS database, enabling capture of Latin American and Portuguese-language studies that are absent from prior reviews. Third, we generated structured evidence gap maps by both measurement construct (attitudes, stereotypes, cognition) and clinical setting, a methodological advance not present in earlier reviews. Fourth, our search extended to November 2025, capturing a growing post-pandemic body of literature on bias in healthcare delivery. Together, these features allow us to provide a more granular and current picture of the field than previously available.

This scoping review synthesizes the growing body of literature examining implicit bias among healthcare professionals, with an emphasis on constructs (attitudes, stereotypes, and cognition), bias targets (e.g., race, gender, weight), and care settings. Our evidence maps highlight race/ethnicity as the most frequently studied bias type, particularly in relation to attitudes, followed by weight and sexual orientation. The predominance of racial/ethnic bias research aligns with prior reviews [23,135] and reflects global calls to address structural racism in healthcare [22,26].

Consistent with previous reviews [8,31], the Implicit Association Test (IAT) remains the most widely used instrument, despite ongoing concerns about its validity and reliability [4,16,136]. In our included references, fewer than half (Table 2 and Table 3; Supplementary Table S6) reported psychometric properties of the tools used. This undermines the interpretability of findings, particularly in studies measuring changes in bias post-intervention [18,29]. As previously noted, IAT scores may be influenced by contextual factors [24], and lack of standardization in administration can hinder cross-study comparisons. Additionally, we found methodological challenges associated with D-score interpretation; some papers mention a potential adaptation or familiarization of participants with the test [12,15,37], which can change the reaction times and affect the detection of truly automatic responses, the theoretical paradigm of the implicit bias assessment. Based on these results, future projects must consider the construction of such differential tools focused on automatic and more authentic non-controlled responses.

Measurement choices were closely tied to the constructs evaluated: stereotype-based studies often used scenario-based tools or vignettes [69,127], while attitudes were mostly assessed via IAT [42,91]. Only a minority of studies incorporated both explicit and implicit measures, potentially limiting the depth of interpretation. This pattern suggests a gap in integrating multi-dimensional approaches to bias, as recommended by Blair (2013) [10] and De Houwer (2019) [9].

Qualitative components were included in several studies [43,78,79], offering insights into context-specific manifestations of bias and its perceived impact on care delivery. However, such mixed-methods designs were rare, even though they can enhance understanding of how bias is enacted in real-world interactions [17,28]. Few studies [44,45,46,47,48,107,127] explored longitudinal outcomes or behavior-based consequences, limiting our ability to assess how biases translate into disparities in health outcomes.

Additionally, gaps persist in the examination of bias across diverse identities. While race/ethnicity remains central, biases related to age, disability, or intersecting identities are underexplored, despite their relevance to healthcare delivery [19,21]. This gap limits the comparability of the measures across different settings and reinforces the importance of developing and adapting tools to assess bias with reliability and validity tests in research and clinical practice. The dominance of academic and hospital settings (See Evidence Maps, Figure 2 and Figure 3) may further restrict the generalizability of findings to primary care or community contexts where different power dynamics are at play.

Finally, our findings underscore the need for theoretical clarity. Some studies failed to specify whether bias was conceptualized as unconscious prejudice, stereotype activation, or judgment distortion. As Holroyd and Puddifoot (2019) [13] and Welpinghus (2020) [5] argue, conceptual ambiguity impedes progress in understanding the mechanisms and potential mitigations of implicit bias.

This scoping review has some limitations. The search strategy relied on selected databases and grey literature sources, which may have omitted relevant studies. Specifically, the absence of a dedicated search in psychology-specific databases such as PsycINFO or APA PsycArticles may have resulted in the omission of relevant studies published primarily in psychology or behavioral science journals, given the inherently interdisciplinary nature of implicit bias research. Additionally, while 104 records could not be retrieved despite two systematic retrieval attempts (inter-library loan and direct author contact), these inaccessible articles, many from lower-income country journals with limited open-access may include relevant findings, particularly from under-represented global regions. Additionally, the heterogeneity of instruments and reporting practices limited comparability across studies. As this was a scoping review, we did not conduct a formal critical appraisal of included sources, which may affect interpretation of the evidence.

## 5. Conclusions

This review highlights a growing but uneven body of research on implicit bias in healthcare. Most studies focus on racial and ethnic bias, often measured through the IAT, with limited attention to other identities, care settings, or behavioral outcomes. Methodological challenges including underreported psychometrics and construct ambiguity, limit comparability and practical application. Broader conceptual frameworks, improved measurement tools, and greater attention to diverse contexts and populations are needed to inform effective mitigation strategies and advance equity in clinical education and practice.

## Figures and Tables

**Table 1:** Basic Characteristics of included studies.

Bias(es) Studied		
Race/Ethnicity	28	[8,42,43,44,45,46,47,48,49,50,51,52,53,54,55,56,57,58,59,60,61,62,63,64,65,66,67,68]
Specific Diseases *	15	[44,69,70,71,72,73,74,75,76,77,78,79,80,81,82]
Multiple Bias	14	[30,78,79,80,81,82,83,84,85,86,87,88,89,90]
Weight	10	[91,92,93,94,95,96,97,98,99,100]
Sexual Orientation	7	[25,101,102,103,104,105,106]
Gender	7	[107,108,109,110,111,112,113]
Disability	5	[114,115,116,117,118]
Other **	4	[119,120,121,122]
No specific	1	[123]
Age	2	[124,125]
**Country of study**		
USA	52	[8,25,43,44,45,46,49,50,51,52,53,54,55,56,58,59,60,61,62,63,64,65,66,67,68,73,74,75,76,78,79,82,84,85,86,87,88,89,90,95,96,97,98,100,101,103,105,111,113,117,118,120]
Canada	9	[42,57,77,83,94,106,122,123,126]
United Kingdom	6	[47,92,104,114,127,128]
Netherlands	3	[99,108,128]
Spain	2	[102,125]
Turkey	2	[93,115]
Compared countries	2	[85,119]
Saudi Arabia	2	[112,129]
Greece	2	[48,130]
India	2	[107,109]
Australia	2	[81,91]
NR	1	[116]
Egypt	1	[69]
Sweden	1	[131]
Germany	1	[70]
Malaysia	1	[106]
Nicaragua	1	[110]
Brazil	1	[121]
Italy	1	[71]
Switzerland	1	[30]
Japan	1	[72]
**Profession of participants**		
Healthcare Professionals	13	[43,56,58,74,80,81,86,98,99,100,114,117,131]
Medical students	13	[25,54,63,70,77,78,87,88,95,104,107,109,125]
Healthcare professionals related with specific condition	10	[59,61,73,75,79,80,92,127,132,133]
Healthcare Students	7	[55,91,103,105,110,122,129]
Physicians	7	[25,56,69,106,108,109]
Primary Healthcare Professionals	7	[65,66,67,76,104,112,118]
Nurses	5	[48,72,90,97,123]
Nurses students	4	[44,82,93,101]
Mental Health Professionals	4	[30,83,102,119]
Surgeons	3	[47,89,113]
Unclear	3	[47,89,113]
Emergency Healthcare professionals	3	[64,84,111]
Therapists	2	[94,116]
Pharmacists	2	[42,120]
Genetic counselors	1	[51]
Dental students	1	[134]
Hospital Healthcare Professionals	1	[130]
Not reported	1	[115]
Social services providers	1	[126]
Oncologists	1	[52]
Ambulance personnel/Paramedics	1	[71]
ICU Healthcare professionals	1	[4]
Chiropractic students	1	[96]
OB-GYNs	1	[53]

* Specific diseases include HIV, sickle cell disease, diabetes, drug addiction, and mental illness. ** Other includes poverty, other health professionals, mental health in general, and sexually transmitted and blood-borne infection bias in general.

**Table 2:** Implicit measurement instruments: administration, scoring, and psychometric reporting.

Instrument	Domains/Subscales	Questions/Items	Assessment Procedure	Bias Assessed	Scoring and Administration Details	Psychometric Reference
IAT	Gender	N/A	Computer-based IAT following standard protocol (Greenwald et al., 1998) [32]. Participants rapidly categorize words or images representing target groups and evaluative attributes using two keys. Reaction time differences between congruent and incongruent pairings are used to calculate the D-score, which indicates implicit bias.	Preference for male professionals, underestimation of women in leadership, or stereotypical career-family associations in healthcare settings	Performance is expressed as the IAT D-score, which quantifies the magnitude and direction of implicit bias. Higher positive or negative D-scores indicate stronger associations favoring one group over another.	Interpretation of IAT D-score values is based on international standards established by Greenwald, Nosek, and Banaji (2003) [15]. The D-score quantifies implicit bias as the difference in reaction times between congruent and incongruent pairings. The IAT is widely validated, but there is no universally accepted cutoff for bias strength; interpretation should be contextualized to the study population and setting. None of the included studies explicitly reported references or detailed psychometric properties for the D-score. Therefore, this methodological reference is provided to support interpretation and reporting in accordance with best scientific practices.
	Disability			Assumptions about limited competence, social exclusion, or reduced expectations in clinical care.		
	Weight			Negative attitudes toward patients with overweight/obesity, beliefs about personal responsibility for weight, or reduced empathy in treatment.		
	Race/Ethnicity			Preference for White over minority patients, stereotypes affecting diagnostic or treatment decisions, or bias in clinical encounters.		
	Sexual Orientation/LGBTQI+			Less positive attitudes toward LGBTQI+ individuals, discomfort in clinical interactions, or expectations of non-traditional family structures.		
	Age			preference for younger over older adults, stereotypes about cognitive decline, or assumptions about capacity for recovery.		
	Disease-specific			Sigma toward people living with HIV, diabetes, or Substance Abuse		
	Multiple Bias/Mixed			Simultaneous assessment of gender and race, or evaluation of bias across several social identities within the same study. This type of implicit Bias, was reported in Systematic Review studies included		
D-PIB (Diabetes Provider Implicit Bias)	No subscales reported.	NR	Computerized performance-based task assessing implicit bias toward patients with Type 1 diabetes. Administration details not fully reported in the included study.	Implicit bias toward pediatric patients with Type 1 diabetes affecting technology recommendations.	Scoring details NR in the included study.	Addala et al. (2021) [73]
IRAP (Implicit Relational Assessment Procedure)	No subscales reported	NR	Response-time paradigm measuring the relative ease of responding to stimulus-response combinations across trial types. Reported in a narrative review of measurement approaches across medical education career stages.	Multiple bias targets in LGBTQ+ health education context.	Response latency differences between pro-true and pro-false trial types indicate implicit relational responding. Full scoring details NR in the included study.	Crump et al. (2025) [88]

NR = Not reported in the included study or in the instrument’s original validation reference. N/A = Not applicable. This table includes only performance-based implicit measures identified across the 93 included studies. For frequency of all instruments identified (implicit and explicit), see Table 3. For full study-level detail, see Supplementary Table S6.

**Table 3:** Frequency of measurement instruments identified across included studies and psychometric reporting.

Instrument	Type	n Studies	Bias Assessed	Psychometric Properties Reported in Included Studies
IAT and variants (BIAT, SC-IAT, BiasProof)	Performance-based implicit	40	Race/ethnicity; weight; gender; sexual orientation; disability; age; disease-specific; multiple	Rarely reported at sample level; D-score interpretation referenced to Greenwald et al., 2003 [15]
Clinical vignettes (various designs)	Scenario-based	8	Race/ethnicity; weight; sexual orientation; multiple	Varies by study; most did not report formal psychometric properties
Beliefs About Obese Persons Scale (BAOP)	Explicit self-report	3	Weight bias	NR in included studies
Health Care Provider HIV/AIDS Stigma Scale (HPASS)	Explicit self-report	3	HIV/AIDS stigma	Rasch analysis reported in one included study [75]
Opening Minds Scale for Health Care Providers (OMS-HC)	Explicit self-report	3	Mental illness stigma	Internal consistency (α ≥ 0.70) reported in some included studies
Attitudes Toward Obese Persons Scale (ATOP)	Explicit self-report	2	Weight bias	NR in included studies
Antifat Attitudes Questionnaire (AFA)	Explicit self-report	2	Weight bias	NR in included studies
D-PIB (Diabetes Provider Implicit Bias tool)	Performance-based implicit	1	Diabetes-related bias	NR in included study
IRAP (Implicit Relational Assessment Procedure)	Performance-based implicit	1	Multiple	NR in included study
Genderism and Transphobia Scale (GTS)	Explicit self-report	1	Gender identity/transphobia	NR in included study
Mental Illness Clinician’s Attitudes Scale (MICA-4)	Explicit self-report	1	Mental illness stigma	NR in included study
Other instruments (*n* = 31)	Explicit self-report/mixed	1 each	Various	See Supplementary Table S6
Total		93 *		

* Studies may report more than one instrument; total reflects number of instrument-study pairs. NR = Not reported. Performance-based implicit measures are described in detail in Table 2. Full instrument-level detail for all explicit measures is available in Supplementary Table S6.

## Data Availability

All data generated or analyzed during this scoping review are included in this published article and its Supplementary Materials.

## Supplementary Materials

The following supporting information can be downloaded at: https://www.mdpi.com/article/10.3390/ijerph23070840/s1, Tables S1–S4: Search strategies for PubMed, Embase, BVS/LILACS, and Google Scholar. Table S5: Excluded studies with reasons for exclusion. Table S6: Characteristics of included studies. Table S7: PRISMA Extension for Scoping Reviews (PRISMA-ScR) Checklist.

## Abbreviations

The following abbreviations are used in this manuscript:

IAT	Implicit Association Test
BIAT	Brief Implicit Association Test
SC-IAT	Single-Category Implicit Association Test
GNAT	Go/No-Go Association Task
AMP	Affect Misattribution Procedure
JBI	Joanna Briggs Institute
PRISMA-ScR	Preferred Reporting Items for Systematic Reviews and Meta-Analyses extension for Scoping Reviews
