# Supplementary material for: Implicit Bias in Health Professionals: A Scoping Review
Source: Int J Environ Res Public Health. 2026 Jun 26;23(7):840. doi: 10.3390/ijerph23070840 (PMC13410340; doi:10.3390/ijerph23070840)
Supplement: Supplementary file 1 [file ijerph-23-00840-s001.zip › ijerph-4210094-supplementary.pdf]

Supplementary File S1.

Search Strategy

Table S1

Component	Terms/Descriptors
**Population**	**MeSH Term:** Health Personnel Other terms: Health workers, Healthcare professionals
**Concept**	**MeSH Term:** Bias, Implicit<br>**Entry Terms:** Subconscious Bias, Bias, Subconscious, Implicit Bias, Hidden Bias, Bias, Hidden, Unconscious Bias, Bias, Unconscious
**Database**	PubMed
**Search String**	(((health personnel[Title/Abstract]) OR (healthcare professional[Title/Abstract])) OR (healthcare[Title/Abstract])) AND ((((stereotyping[Title/Abstract]) OR (prejudice[Title/Abstract])) OR (attitudes of health personnel[Title/Abstract])) OR (implicit bias[Title/Abstract]))
**Search Date**	August 24, 2023<br>Update: November 2025

Table S2

Component	Terms/Descriptors
**Population**	'health care personnel':ab,ti OR 'health care':ab,ti
**Concept**	'implicit bias':ab,ti OR 'attitudes of health personnel':ab,ti OR prejudice:ab,ti

**Search Combination**	#1 = Population terms<br>#2 = Intervention terms<br>#1 AND #2
**Database**	Embase
**Search String**	('health care personnel':ab,ti OR 'health care':ab,ti) AND ('implicit bias':ab,ti OR 'attitudes of health personnel':ab,ti OR prejudice:ab,ti)
**Search Date**	August 24, 2023<br>Update: November 2025

Table S3

Component	Terms/Descriptors
**Population**	"health care personnel" OR "health care"
**Concept**	"implicit bias" OR "attitudes of health personnel" OR "prejudice"
**Database**	BVS - LILACS
**Search String**	("health care personnel" OR "health care") AND ("implicit bias" OR "attitudes of health personnel" OR prejudice)
**Search Date**	August 24, 2023<br>Update: November 2025

Table S4

Component	Terms/Descriptors
**Population**	"health care personnel" OR "health care"
**Concept**	“implicit bias” OR attitudes OR prejudice

**Database**	Google Scholar
**Search String**	"health care personnel" AND (“implicit bias” OR attitudes OR prejudice)
**Search Date**	August 24, 2023<br>Update: November 2025

Table S5. Excluded studies

Author year	Title	Reason
Sally 2021	Gaps in Measuring and Mitigating Implicit Bias in Healthcare	Not relevant to the review question
Dias 2012	Health workers' attitudes toward immigrant patients: a cross-sectional survey in primary health care services	Not relevant to the review question
Weber 2020	Country-specific differences of age stereotypes towards older hospital staff and their association with self-efficacy, work ability and mental well-being	Not relevant to the review question
Gupta 2020	Discrimination in the health care system among higher-weight adults: evidence from a Canadian national cross-sectional survey	Not relevant to the review question
Vela 2022	Eliminating Explicit and Implicit Biases in Health Care: Evidence and Research Needs	Not relevant to the review question
Pennington 2023	A mixed-methods evaluation of unconscious racial bias training for NHS senior practitioners to improve the experiences of racially minoritised students.	Not relevant to the review question
Singer 2023	Improving the Knowledge, Attitudes, and Behavioral Intentions of Perinatal Care Providers Toward Childbearing Individuals Identifying as LGBTQ: A Quasi-Experimental Study	Not relevant to the review question
Strauss 2023	Multicenter study of racial and ethnic inequities in liver transplantation evaluation: Understanding mechanisms and identifying solutions	Not relevant to the review question
Schwartz 2019	Implicit bias training in emergency medicine residency: There's a right answer	Not relevant to the review question
Burke 2017	Medical students' knowledge of and beliefs about LGBT and their health care needs: What's the impact of a class on LGBT adolescent health?	Not relevant to the review question
Ayhan 2019	Perceived prejudice in healthcare and women's health protective behavior	Not relevant to the review question
Miller 2000	Oncology healthcare professionals' attitudes toward elderly people.	Not relevant to the review question
Rauf 2022	Conscious and Unconscious Bias: The Hidden Pandemic of Biases in Healthcare Exacerbated by COVID-19	Not relevant to the review question
Oliveira 2020	"Experiences marked by prejudice(s)?": nurses' representations on 'transvestite' people	Not relevant to the review question

Pearl 2018	Ageism and Health in Patients Undergoing Cosmetic Procedures.	Not relevant to the review question
Banwari 2015	Medical students and interns’ knowledge about and attitude towards homosexuality	Not relevant to the review question
Solola 2020	Race and Gender-Based Perceptions of Older Adults: Will the Youth Lead the Way?	Not relevant to the review question
Xie 2019	On the Bias of Precision Estimation Under Separate Sampling	Not relevant to the review question
Balsa 2005	Testing for Statistical Discrimination in Health Care	Not relevant to the review question
Miller 2018	How Do I Respond to Cultural Biases?	Not relevant to the review question
Schwartz 2020	Fostering Empathy, Implicit Bias Mitigation, and Compassionate Behavior in a Medical Humanities Course	Not relevant to the review question
Mobula 2015	Cultural competence and perceptions of community health workers' effectiveness for reducing health care disparities	Not relevant to the review question
Alsalem 2018	Assessing safety climate in acute hospital settings: a systematic review of the adequacy of the psychometric properties of survey measurement tools	Not relevant to the review question
Burton 2021	Development of the Perception of Opioid Use Survey Instrument	Not relevant to the review question
Beneduce 2005	Politics of healing and politics of culture: ethnopsychiatry, identities and migration	Not relevant to the review question
Warne 2008	Disorders of sex development (DSDs), their presentation and management in different cultures	Not relevant to the review question
Sagbakken 2018	Dementia and Migration: Family Care Patterns Merging With Public Care Services	Not relevant to the review question
Dalton 2018	Factors that influence nurses' assessment of patient acuity and response to acute deterioration	Not relevant to the review question
Carvalho 2021	Why does your pain never get better? Stigma and coping mechanism in people with sickle cell disease	Not relevant to the review question
Harrison 2019	Beliefs and challenges held by medical staff about providing emergency care to migrants: an international systematic review and translation of findings to the UK context	Not relevant to the review question

Bowler 1993	Stereotypes of women of Asian descent in midwifery: some evidence	Not relevant to the review question
Yamadan 2023	Perceptions toward issues in cancer care for people with mental illness among psychiatric care providers: A questionnaire study	Not relevant to the review question
DiDomizio 2023	Challenges to Achieving HCV Micro-Elimination in People With HIV in the United States: Provider Perspectives and the Role of Implicit Bias	Not relevant to the review question
Thompson 2023	Educational strategies in the health professions to mitigate cognitive and implicit bias impact on decision making: a scoping review	Not relevant to the review question
Thi 2022	Socio-cultural Norms and Gender Equality of Ethnic Minorities in Vietnam	Not relevant to the review question
Simmons 2022	Characteristics Associated With Being Asked About Violence Victimization in Health Care: A Swedish Random Population Study	Not relevant to the review question
Ogunyemi 2021	Defeating Unconscious Bias: The Role of a Structured, Reflective, and Interactive Workshop	Not relevant to the review question
Mulchan 2021	What COVID-19 Teaches Us About Implicit Bias in Pediatric Health Care	Not relevant to the review question
Saluja 2021	How Implicit Bias Contributes to Racial Disparities in Maternal Morbidity and Mortality in the United States	Not relevant to the review question
Acholonu 2020	Interrupting Microaggressions in Health Care Settings: A Guide for Teaching Medical Students	Not relevant to the review question
Tate 2020	Evaluating the effectiveness of a structural competency and bias in medicine curriculum for internal medicine residents	Not relevant to the review question
Brottman 2020	Toward Cultural Competency in Health Care: A Scoping Review of the Diversity and Inclusion Education Literature	Not relevant to the review question
Perdomo 2019	Health Equity Rounds: An Interdisciplinary Case Conference to Address Implicit Bias and Structural Racism for Faculty and Trainees	Not relevant to the review question
Teall 2019	Faculty Perceptions of Engaging Students in Active Learning to Address Implicit Bias Using Videos Exemplifying the Prenatal Visit of a Lesbian Couple	Not relevant to the review question
Oatis 2019	41.2 “CAN WE TALK?” POLITICS, SEXUALITY, GENDER, AND RACE	Not relevant to the review question
Morris 2019	Training to reduce LGBTQ-related bias among medical, nursing, and dental students and providers: a systematic review	Not relevant to the review question

Narayan 2019	CE: Addressing Implicit Bias in Nursing: A Review	Not relevant to the review question
Weech-Maldonado 2018	Hospital cultural competency as a systematic organizational intervention: Key findings from the national center for healthcare leadership diversity demonstration project	Not relevant to the review question
Martinez 2016	A longitudinal psychosocial curriculum to enhance residents' self-efficacy in delivering culturally competent care	Not relevant to the review question
Chung 2013	Perceptions on gender awareness and considerations in career choices of medical students in a medical school in Taiwan	Not relevant to the review question
Müller 2013	Teaching lesbian, gay, bisexual and transgender health in a South African health sciences faculty: addressing the gap.	Not relevant to the review question
Shields 2012	Lesbian, gay, bisexual, and transgender parents seeking health care for their children: a systematic review of the literature.	Not relevant to the review question
Refai 2012	Public beliefs and attitudes about schizophrenia, major depression and psychotropic medication	Not relevant to the review question
Heslehurst 2018	Perinatal health outcomes and care among asylum seekers and refugees: a systematic review of systematic reviews.	Not relevant to the review question
Elias 2021	The Costs of Institutional Racism and its Ethical Implications for Healthcare.	Not relevant to the review question
Torres 2022	Implicit biases in healthcare: implications and future directions for gynecologic oncology.	Not relevant to the review question
Morrison 2022	Bias and discrimination in surgery: Where are we and what can we do about it?	Not relevant to the review question
Kidd 2022	A survey of implicit bias training in physician assistant and nurse practitioner postgraduate fellowship/residency programs.	Not relevant to the review question
Salguero 2019	Is there an association between ageist attitudes and frailty?	Not relevant to the review question
Shangani 2022	Cultural adaptation and validation of a measure of prejudice against men who have sex with men among healthcare providers in western Kenya.	Not relevant to the review question
Shapiro 2018	Implicit Physician Biases in Periviability Counseling.	Not relevant to the review question
Shangani 2022	Sexual Prejudice and Comfort to Provide Services to Men Who Have Sex with Men Among HIV Healthcare Workers in Western Kenya: Role of Interpersonal Contact.	Not relevant to the review question

MesquitaFilho 2018	Sexism against women among primary healthcare workers.	Not relevant to the review question
OzelBilim 2021	The psychometric properties, confirmatory factor analysis, and cut-off value for the Fraboni scale of ageism (FSA) in a sampling of healthcare workers.	Not relevant to the review question
Chapman 2018	Making a difference in medical trainees' attitudes toward Latino patients: A pilot study of an intervention to modify implicit and explicit attitudes.	Not relevant to the review question
Smith 2022	It's not all White: Implicit Racial Bias in Imagery Used in Plastic Surgery Resident Education.	Not relevant to the review question
Huang 2023	Racial Disparities in Barriers to Care for Patients With Diabetic Retinopathy in a Nationwide Cohort.	Not relevant to the review question
DominicÃ©Dao 2018	Vulnerability in the clinic: case study of a transcultural consultation.	Not relevant to the review question
Ufomata 2018	Comprehensive Internal Medicine Residency Curriculum on Primary Care of Patients Who Identify as LGBT.	Not relevant to the review question
Elenwo 2023	Racial discrimination among children in the United States from 2016 to 2020: an analysis of the National Survey of Children's Health.	Not relevant to the review question
Coleman 2017	Sexual Orientation Disclosure in Primary Care Settings by Gay, Bisexual, and Other Men Who Have Sex with Men in a Canadian City.	Not relevant to the review question
Cohen 2019	Influence of weight etiology information and trainee characteristics on Physician-trainees' clinical and interpersonal communication.	Not relevant to the review question
Harris 2017	Measuring the bias against low-income country research: an Implicit Association Test.	Not relevant to the review question
Oishi 1983	[Study on hospice movements. A survey on the attitudes of health personnel toward patients in the terminal stage of cancer]	Language
Marek 2020	[Prejudices and their healthcare implications. Lessons learnt from a national survey].	Language
Chen 2023	[The Intrinsic Problem of Labeling in Healthcare].	Language
Rosenbloom 2019	Healthcare provider perceptions of disparities in perioperative care	Wrong intervention
Ogden 2020	Recruiting and retaining general practitioners in rural practice: systematic review and meta-analysis of rural pipeline effects	Wrong intervention

Burgess 2017	Mindfulness practice: A promising approach to reducing the effects of clinician implicit bias on patients	Wrong intervention
Trent 2019	Please Be Careful with Me: Discrepancies between Adolescent Expectations and Clinician Perspectives on the Management of Pelvic Inflammatory Disease	Wrong intervention
Cox 2023	Medical Mistrust Among a Racially and Ethnically Diverse Sample of Sexual Minority Men	Wrong intervention
Wu 2019	The Efficacy of an Antioppression Curriculum for Health Professionals	Wrong intervention
Stone 2011	Non-conscious bias in medical decision making: what can be done to reduce it?	Wrong intervention
Ricks 2022	Undoing Racism and Mitigating Bias Among Healthcare Professionals: Lessons Learned During a Systematic Review.	Wrong intervention
Tajeu 2022	Development of a Multicomponent Intervention to Decrease Racial Bias Among Healthcare Staff.	Wrong intervention
Tajeu 2018	Exploring the Association of Healthcare Worker Race and Occupation with Implicit and Explicit Racial Bias.	Wrong intervention
Ruben 2020	Addressing Implicit Bias in First-Year Medical Students: a Longitudinal, Multidisciplinary Training Program.	Wrong intervention
Tjia 2021	COmmuNity-engaged SimULation Training for Blood Pressure Control (CONSULT-BP): A study protocol.	Wrong intervention
Galiatsatos 2022	A Checklist to Address Implicit Bias in Healthcare Settings During the COVID-19 Pandemic: The PLACE Strategy.	Wrong intervention
Matharu 2014	Reducing obesity prejudice in medical education.	Wrong intervention
Joseph 2023	Feasibility of a Loving Kindness Intervention for Mitigating Weight Stigma in Nursing Students: A Focus on Self-Compassion.	Wrong intervention
Gutierrez 2014	Fair Play: A Videogame Designed to Address Implicit Race Bias Through Active Perspective Taking.	Wrong intervention
Tjia 2023	Using Simulation-Based Learning with Standardized Patients (SP) in an Implicit Bias Mitigation Clinician Training Program.	Wrong intervention
Amsalem 2021	Reducing Stigma Toward Individuals With Schizophrenia Using a Brief Video: A Randomized Controlled Trial of Young Adults.	Wrong intervention

Grailey 2022	Exploring the working environment of Hospital Managers: a mixed methods study investigating stress, stereotypes, psychological safety and individual resilience.	Wrong intervention
Devlin 2020	The Effect of Discrimination on Likelihood of Participation in a Clinical Trial.	Wrong intervention
Roach 2022	Anti-Indigenous bias of medical school applicants: a cross-sectional study.	Wrong intervention
Pettit 2017	Effect of Socioeconomic Status Bias on Medical Student-Patient Interactions Using an Emergency Medicine Simulation.	Wrong intervention
Malterud 2011	Obesity, stigma, and responsibility in health care: A synthesis of qualitative studies	Wrong outcomes
Schlebusch 1991	Health care professionals' knowledge about AIDS, prejudice and attitudes towards AIDS	Wrong outcomes
Koyi 2018	Change in Medical Student Attitudes Toward Patients with Substance Use Disorders After Course Exposure	Wrong outcomes
Sabin 2022	Tackling Implicit Bias in Health Care	Wrong outcomes
Abramson 2015	Perceived discrimination in U.S. healthcare: Charting the effects of key social characteristics within and across racial groups.	Wrong outcomes
Mbuzi 2017	Indigenous peoples' experiences and perceptions of hospitalisation for acute care: A metasynthesis of qualitative studies.	Wrong outcomes
Monaghan 2021	Prevalence and Stigma of Postpartum Common Mental Disorders in the Gurage Region of Ethiopia: A Mixed-Methods Observational Cohort Study.	Wrong patient population
Ayalon 2023	Combatting ageism through virtual embodiment? Using explicit and implicit measures.	Wrong patient population
Meadows 2017	Social Dominance Orientation, Dispositional Empathy, and Need for Cognitive Closure Moderate the Impact of Empathy-Skills Training, but Not Patient Contact, on Medical Students' Negative Attitudes toward Higher-Weight Patients.	Wrong patient population
Willis 2023	The "Intern" Label: Introducing Unnecessary Confusion and Bias?	Wrong patient population
Jindal 2022	Effects of a Curriculum Addressing Racism on Pediatric Residents' Racial Biases and Empathy	Wrong setting
Johnson 2020	Combining SOAP Notes With Guided Reflection to Address Implicit Bias in Health Care	Wrong setting

Thorburn 2005	African American women and family planning services: Perceptions of discrimination	Wrong setting
Fernandes 2022	Addressing Ageism-Be Active in Aging: Study Protocol	Wrong study design
Usama 2023	EXPLORING INFANT FEEDING IMPLICIT BIAS	Wrong study design
Astudillo 2022	FOUNDATION AND DECISION-MAKING FRAMEWORK FOR UNDERSTANDING AND COMBATING IMPLICIT BIAS IN PEDIATRICS	Wrong study design
Odugbesan 2022	DIABETES PROVIDER BIAS TO RECOMMENDING DIABETES TECHNOLOGY FOR PATIENTS ON PUBLIC INSURANCE IN THE UNITED STATES	Wrong study design
Job 2022	Health professional's implicit bias of adult patients with low socioeconomic status (SES) and its effects on clinical decision-making: a scoping review protocol.	Wrong study design
Hausmann 2015	Examining implicit bias of physicians who care for individuals with spinal cord injury: A pilot study and future directions.	Wrong study design
Murrar 2023	Predictors of Perceived Discrimination in Medical Settings Among Muslim Women in the USA.	Wrong study design
Penner 2013	Racial Healthcare Disparities: A Social Psychological Analysis.	Wrong study design
Parildar 2021	Are patients with obesity "scapegoats"? The obesity prejudice levels of health care workers in Turkey	Not relevant to the review question

Table S6. Characteristics of included references

Reference (Author, Year)	Country	Population	Setting	Method for Assessing Bias	Construct	Main Finding Related to Bias
Alzahrani 2022	Saudi Arabia	Primary Healthcare Professionals in Saudi Arabia. The five regions were Riyadh, Madinah, Aseer, Tabouk, and the Eastern Region	Primary care	Implicit bias: The IAT, Implicit Association Tests (IAT) established by Harvard University<br><br>Explicit bias: one single question (non specific instrument)	Attitudes, Cognition	Persistent bias against women in leadership remains in Saudi healthcare despite reforms. Both men and women exhibit implicit and explicit bias, regardless of experience or specialty. Key predictors of bias were identified using mixed measures
Aziz 2021	Egypt	Physicians working at University Hospital	Hospital-based, Academic	Arabic version of the Health Care Provider HIV/AIDS Stigma Scale (HPASS)	Stereotype	The Arabic HPASS was successfully translated and culturally adapted for use in Egypt. It showed strong psychometric validity to assess HIV-related stigma among physicians, supporting future intervention programs
Barradel 2023	United Kingdom	Healthcare professionals related with COPD patients (Doctors, Nurses, Physiotherapists)	Hospital-based, Academic	Implicit Association Test (IAT) facilitated by PsyToolkit's online platform	Attitudes	These results demonstrate the presence of implicit negative bias towards smoking and implicit positive bias towards exercising amongst HCPs who refer individuals with COPD to PR. Due to the correlation between beliefs and behaviour, this provides a rationale for the inclusion of components targeting HCP communication (e.g. decision coaching training, consultation prompt) in our shared decision-making intervention to enable HCPs to fully and impartially support individuals' decision-making for PR.
Bower 2023	USA	Maternal healthcare professionals (physicians, midwives, nurse practitioners, physician assistants, nurses, and patient care technicians)	Primary care, Hospital-based	They developed and validated a new instrument for measuring awareness and mitigation of bias among maternal healthcare professionals: The Bias in Maternal Health Care scale	Cognition	A 3-phase study developed the Bias in Maternal Health Care scale, showing validity and reliability to assess bias awareness and mitigation in maternal healthcare. Higher scores in IBT-exposed participants support its use in evaluating bias training programs and adapting it to other medical fields.

Reference (Author, Year)	Country	Population	Setting	Method for Assessing Bias	Construct	Main Finding Related to Bias
Jayawickrama 2023	Australia	Undergraduate and postgraduate students related to health care at Australian universities	Academic	Implicit Association Test (IAT)<br>Interpersonal Reactivity Index (IRI)<br>EXPLICIT WEIGHT BIAS (4 scales):<br>- AFA: Antifat attitudes questionnaire<br>- BAOP: Beliefs about obese persons scale<br>- Empathy for Obese Patients Scale<br>- Confidence in Clinical Interaction with Obese Patients Scale	Attitudes	Results demonstrate the presence of both explicit and implicit weight bias among Australian health care students. Several characteristics and experiences of students were associated with their weight bias (e.g., Body Mass Index, and experiences such as having witnessed the enactment of weight stigma by role models). We also found that greater empathic concern contributed to lower explicit weight bias. Validity of the exhibited weight bias should be established in practical interactions with people living with overweight or obesity and novel interventions should be developed to ameliorate weight bias.
Ni 2023	Malaysia	Physicians	Hospital-based, Academic	Multidimensional HIV stigma scale (adapted version of the intention-to-discriminate at work subscale)	Stigma	Despite a low average intention to discriminate, over 70% of physicians showed some bias toward MSM in Malaysia. Stigma-related constructs—prejudice, shame, fear—were linked to discriminatory intent. Findings highlight the need for targeted medical education to reduce stigma and improve HIV care access
VanPuymbrouck 2022	USA	health care providers (Occupational and physical therapy assistants, Diagnosis and treating practitioners (MD, dentist, etc.), Technologists and technicians, Nursing and home health assistants and others)	Primary care, Hospital-based, Community	Disability Attitude Implicit Association Test (disability attitudes version of the IAT)<br><br>explicit measure of disability attitudes	Attitudes	83.6% of providers showed implicit bias against people with disabilities (PWD), while most reported no explicit preference. This gap suggests hidden bias among clinicians. Most providers were aversive ableists (low explicit, high implicit). The study identifies demographic factors linked to lower bias, offering targets for bias-reduction in health education and policy.

Reference (Author, Year)	Country	Population	Setting	Method for Assessing Bias	Construct	Main Finding Related to Bias
Salles 2019	USA	Surgeons and other health care professionals	Primary care, Hospital-based, Community	Gender-Career IAT	Attitudes	The data from Project Implicit's Gender-Career IAT suggest that men and women in health care strongly implicitly associate men with career and women with family. With regard to explicit bias, however, men in health care were more likely than women to associate men with career and women with family. These findings are similar to what we found with the Gender-Specialty IAT assessing bias among surgeons. Surgeons tended to associate men with surgery and women with family medicine. The main contribution of this work is an initial estimate of the extent of implicit gender bias within health care. Future research could examine implications of implicit gender biases on gender inequality and discrimination.
Wagner 2017	Canada	Medical and nursing students from across Canada	Academic	The Health Care Provider HIV/AIDS Stigma Scale (HPASS) ; The Color-Blind Racial Attitudes Scale (CoBRAS); The Modern Sexism Scale (MSS); The Homophobia Scale (HS) ; an adapted version of the Sex Worker Stigma Index (SWSI); The Attitudes Toward Injection Drug Use Scale (ATIS)	Attitudes	HPASS scores correlated positively with multiple stigma types, supporting its validity. Canonical correlation (RC = .64) showed 41% shared variance between stigma and HPASS, with prejudice and Stereotypes contributing most. All stigma measures except sexism were significant predictors. Removing sexism improved model fit slightly, suggesting sexism's limited role in explaining stigma in this context.
Addala 2021	USA	Multi-disciplinary pediatric diabetes providers	Primary care, Hospital-based	Diabetes Provider Implicit Bias (D-PIB)	Attitudes, Stereotype	Among 39 providers, most were non-Hispanic White (79.5%), female (89.7%), and worked in urban/academic settings. Providers ranked family preference as the top factor in recommending CGM and insulin pumps. Insurance type ranked second for CGM and fourth for pumps, while race/ethnicity was ranked least important, suggesting implicit bias may not overtly influence device recommendations.
Sabin 2015	International sample (USA, Australia, United Kingdom, Canada)	Health professionals (doctors, nurses and others)	Hospital-based, Primary care	IAT<br><br>Explicit Bias Questionary	Attitudes	Found that moderate to strong implicit preferences for straight people over lesbian women or, in particular, gay men, are widespread among heterosexual providers. In contrast, lesbian and gay providers held implicit and explicit preferences for lesbian women and gay men over straight people, and bisexual providers held mixed preferences. Of provider types, mental health providers generally held the weakest implicit preferences toward heterosexual people, and nurses held the strongest. Heterosexual, lesbian, and gay people in almost all provider groups reported moderate to strong explicit preferences for people who shared their own sexual identity. There was 1 exception: heterosexual female mental health providers

Reference (Author, Year)	Country	Population	Setting	Method for Assessing Bias	Construct	Main Finding Related to Bias
						explicitly reported favoring lesbian women and gay men over heterosexual people.
Burke 2015	USA	Medical students (heterosexual, first-year)	Academic	IAT<br>Explicit bias: Feeling thermometer self-assessments<br>Contact questionaries<br>Empathy: Interpersonal Reactivity Index	Attitudes, Stereotype	Nearly half (45.79%; 956/2,088) of respondents with complete data on both bias measures expressed at least some explicit bias, and most (81.51%; 1,702/2,088) exhibited at least some implicit bias against gay and lesbian individuals. Both amount and favorability of contact predicted positive implicit and explicit attitudes. Both cognitive and emotional empathy predicted positive explicit attitudes, but not implicit attitudes. The prevalence of negative attitudes presents an important challenge for medical education, highlighting the need for more research on possible causes of bias. Findings on contact and empathy point to possible curriculum-based interventions aimed at ensuring high quality care for sexual minorities.
Heather 2021	NR	occupational therapy (OT) and physical therapy (PT)	Primary care, Hospital-based, Academic	IAT	Stereotype	Findings revealed the majority of OTA/PTA reported having no explicit preference for people with disabilities or nondisabled people. However, the majority of OTA/PTA were aversive ableists, indicating low explicit and high implicit bias.
Burgess 2014	USA	Primary care physicians from the Veterans Affairs Healthcare System	Primary care	Clinical Vignette	Attitudes, Stereotype	A three-way interaction showed that prescribing decisions varied by patient race, physician gender, and cognitive load (p = 0.034). Under high cognitive load, male physicians prescribed fewer opioids to Black vs. White patients (12.5% vs. 30%), while female physicians prescribed more to Black patients (39.1% vs. 15.8%). Patterns reversed or narrowed under low cognitive load.
Nathan 2019	USA	Genetic Counselors' and Genetic Counseling Students	Primary care, Hospital-based, Community	®Sexuality Implicit Association Test (SIAT) and the Explicit Attitudes and Demographics Questionnaire	Attitudes, Stereotype	In a web-based survey of 575 participants, 60.2% showed implicit preference for heterosexual over homosexual people, despite low explicit bias. Implicit bias varied by sexual orientation but not by gender, race, or specialty. Most participants (95.8%) supported incorporating LGBT-related content into genetic counseling education, highlighting its potential to reduce bias.
Bean 2013	USA	nursing and medical students	Primary care, Hospital-based, Community	nursing and medical students	Attitudes, Stereotype	This study found that both nursing and medical students exhibited nonconscious Stereotype activation toward Hispanic patients, associating them more readily with noncompliance and health risk terms. The effect was independent of participants' explicit motivation to control bias. Results highlight the importance of incorporating strategies to reduce implicit stereotyping into cultural competence training.

Reference (Author, Year)	Country	Population	Setting	Method for Assessing Bias	Construct	Main Finding Related to Bias
Lowe 2020	USA	Unclear	Primary care, Hospital-based, Community	Implicit Association Test (IAT)	Stereotype	Genetic counselors (GCs) used more cognitive facilitation strategies with racial/ethnic minority clients than with White clients. However, no significant association was found between GCs' pro-White implicit bias and facilitation of clients' cognitive or emotional processing. Results suggest implicit bias may not influence these specific communication behaviors, or that measurement tools may lack sensitivity to detect such effects.
Pleuhs 2022	USA	healthcare providers practicing in the 10 U.S. citie	Primary care, Hospital-based, Community	several patient vignettes	Attitudes, Stereotype	Among 480 providers, 82.6% would prescribe PrEP to a PWID vignette patient, but 13% were unsure and 4.4% said no. Lack of knowledge (42.9%) and adherence concerns (27.4%) were key barriers. A minority (7.1%) showed explicit bias, despite the patient clearly meeting CDC PrEP criteria. Overall, findings highlight persistent stigma and provider discomfort, particularly toward people who inject drugs (PWID), despite PrEP's proven effectiveness. Provider training and systemic interventions are needed to overcome barriers and reduce bias in HIV prevention care.
Wagner 2017	Canada	Health care and social service providers	Primary care, Hospital-based, Community	STBBI Stigma Scale	Attitudes, Stereotype	The new scale, consisting of 21 items for each category, demonstrated excellent internal consistency, reliability, and convergent and divergent validity. The factor structure of the scale supports a tripartite model of stigma consisting of stereotyping, prejudice and discrimination. Stereotyping had the highest relative scores on the subscales, and attitudes regarding other viral STBBIs differed significantly from the other STBBI categories.
Puumala 2017	USA	physicians, nurses and advanced practice providers at five EDs in the Upper Midwest	Primary care, Hospital-based, Community	Implicit Association Test (IAT), Vignettes	Stereotype	A total of 154 care providers completed the survey. Agreement with negative American Indian Stereotypes was 22–32%. Overall, 84% of providers had an implicit preference for non-Hispanic white adults or children. Older providers (≥ 50 years) had lower implicit bias than those middle aged (30–49 years), ($p = 0.01$). American Indian children were seen as increasingly challenging ($p = 0.04$) and parents/caregivers less compliant ($p = 0.002$) as the proportion of American Indian children seen in the ED increased. Responses to the vignettes were not related to implicit or explicit bias.
Ohm 2011	USA	Baccalaureate Student Nurses	Academic	Perceived Prejudice in Health Care Scale–Modified (PPHC-M)	Stereotype	The PPHC-M scale showed good reliability and construct validity. While the overall perception of prejudice in healthcare was neutral (mean = 0.296), subgroup analysis revealed a strong perception of prejudice among non-White students (mean = 5.1). The results suggest

Reference (Author, Year)	Country	Population	Setting	Method for Assessing Bias	Construct	Main Finding Related to Bias
						demographic factors (race, age, education) influence perceptions of prejudice, and highlight the need for targeted sampling and future validation in diverse populations. Social desirability bias may also lead to underreporting prejudice, especially in younger or predominantly White samples.
Roach 2023	Canada	active physicians	Primary care	Survey and feeling thermometer approaches (Explicit anti-Indigenous bias) .	Attitudes, Stereotype	Explicit and implicit anti-Indigenous bias were identified among healthcare providers. While most reported favorable feelings toward Indigenous people (median = 84/100), 8.3% expressed unfavorable attitudes, and 25% showed a preference for white people. Implicit bias scores revealed a moderate overall preference for white faces (median IAT = −0.34). Cisgender men and White cisgender men showed the highest levels of implicit bias. Remote practitioners exhibited the most neutral biases, while those in urban/surgical settings and older providers showed stronger pro-white bias. Findings underscore the presence of both explicit and implicit anti-Indigenous bias, especially in certain demographics.
Abiola 2021	USA	Healthcare Professional Population: The article discusses the impact of unconscious bias among healthcare professionals, including nurses, physicians, and public health workers.	Primary care, Hospital-based, Community	The article reviews existing literature and expert opinions on unconscious bias but does not specify a single assessment too	Attitudes, Stereotypes, Cognition	Unconscious bias among healthcare professionals contributes to disparities in treatment and health outcomes for Black, Indigenous, and other non-white communities. Implicit biases can influence clinical decisions, leading to inequitable care. Addressing unconscious bias requires systemic changes, including training, policy reforms, and a commitment to health equity.
Suri 2024	USA	Leaders in emergency medicine (EM), including attending physicians,	Hospital-based, Academic	qualitative, arts-based methods	Stereotype	A total of 125 responses were analyzed. Four major themes emerged: (1) acceptance that bias exists; (2) individual barriers, including fear of negative reactions,often due to power dynamics between respondents and other members of the ED; (3)institutional barriers, such as insufficient funding and unprotected time committed to addressing bias; and (4) ambiguity about defining and prioritizing bias.

Reference (Author, Year)	Country	Population	Setting	Method for Assessing Bias	Construct	Main Finding Related to Bias
		residents/fellows, and medical students, primarily affiliated with the Society for Academic Emergency Medicine (SAEM) Leadership Academy. The participants consisted of 62 attending physicians, eight residents/fellows, and four medical students.				
Cleveland 2020	USA	faculty members in emergency medicine	Hospital-based, Academic	Implicit Association Tests (IATs)	Stereotype	Among EM faculty, 59% demonstrated implicit gender bias associating males with science—more common among men (67%) than women (40%). On the Race IAT, 45% showed implicit preference for White individuals, compared to 20% for Black individuals. Agreement with IAT results was higher for gender (57%) than for race (40%). Female faculty more often perceived unconscious bias in faculty recruitment, promotion, and leadership opportunities than males (all $p < 0.005$). Compared to the general population, faculty showed less implicit bias overall, yet notable levels of bias persist.

Reference (Author, Year)	Country	Population	Setting	Method for Assessing Bias	Construct	Main Finding Related to Bias
Őri 2023	Albania, Austria, Azerbaijan, Belarus, Belgium, Bulgaria, Croatia, Czech Republic, Denmark, Estonia, France, Germany, Greece, Hungary, Ireland, Italy, Latvia, Lithuania, Montenegro, Netherlands, Portugal, Russia, Serbia, Slovakia, Slovenia, Spain, Switzerland, Turkey, Ukraine, United Kingdom	Practising specialists in general adult psychiatry, Trainees in general adult psychiatry, Practising specialists in child and adolescent psychiatry, Trainees in child and adolescent psychiatry	Primary care, Hospital-based, Academic	Opening Minds Stigma Scale for Health Care Providers (OMS-HC)	Attitudes	Psychiatrists (n=4245): Less stigma linked to personal experience with mental illness, supportive work environments, providing psychotherapy, and participating in reflective practices (e.g., case discussions, Balint groups). All factors significantly associated with reduced stigma ($p < 0.05$).
Wada 2016	Japan	Nurses currently working in hospitals and clinics	Hospital-based	survey tool	attitudes, Stereotype	Nurses (n=992, mostly women 30–49): 53% had HIV-related anxiety; 54% for HBV/HCV. Reluctance to care: 41% (HIV), 18% (HBV/HCV). Prejudice higher for HIV (39%) vs HBV/HCV (13%). Reluctance linked to anxiety (HIV OR: 3.58; HBV/HCV OR: 6.36), reduced by confidence and prior experience. Age >50 linked to HIV care reluctance (OR: 1.21).
Hopp 2023	Germany	fourth-year medical students who attended a week-long practical block on	Academic	the German version of the Mental Illness: Clinician's attitudes Scale 4 (MICA-4), The students' global self-worth and self-generated questionnaire	Attitudes, Stereotype	Medical students: Stigma toward mental illness decreased more with psychiatric patient exposure vs. somatic instruction ($p = .019$, $\eta^2 = .04$). Female gender, prior contact, and interest in psychiatry linked to lower stigma; males and those with low self-esteem showed higher initial stigma. No moderators of change identified.

Reference (Author, Year)	Country	Population	Setting	Method for Assessing Bias	Construct	Main Finding Related to Bias
		psychosomatic medicine				
Etienne 2024	USA	students currently enrolled in a pre-licensure nursing program and completion of at least one semester of clinical experience in a hospital setting	Academic	focus group, semi-structured questions	Stereotype	Three themes on racial implicit bias (RIB) in nursing emerged: (1) recognition of bias without formal terminology or training; (2) tension between RIB behaviors and professional nursing ethics; and (3) acknowledgment of RIB's negative impact on patient outcomes, highlighting gaps in education and institutional accountability.
Howard 2024	USA	All clinicians that deliver vascular care in the United States (US) are eligible to participate in the VQI	Academic	Race implicit association test (IAT), Bias Awareness Measure	Stereotype	There were 2,512 surgeons in the VQI registry, 304 of whom completed the survey, including getting IAT results. Most participants (71.6%) showed a pro-White bias with 73.0% of this group in the moderate and strong categories. While 77.5% of respondents showed conscious awareness of bias, of those whose conscious results showed lack of awareness, 67.8% had moderate or strong bias, compared to 55.7% for those with awareness. Bias magnitude varied based on physician race/ethnicity and years of experience. Women were more likely than men to report awareness of biases and potential impact of bias on decision-making.
Turner 2024	USA	pediatric HCPs who are licensed as physicians, physician assistants (PAs), registered nurses (RNs), licensed practical nurses (LPNs), and advanced practice nurses; provide care to children ages 18 and under; and have access to a	Community, Hospital-based, Primary care	Beliefs About Weight Stigma Questionnaire (BAWSQ), IAT	Attitudes, Stereotype	Participants showed moderate-to-high implicit and explicit weight bias. Implicit bias was associated with years in practice and occupation ($p < 0.05$), while explicit bias correlated with several items, indicating that providers aware of their weight bias may be willing to address it.

Reference (Author, Year)	Country	Population	Setting	Method for Assessing Bias	Construct	Main Finding Related to Bias
		desktop or laptop computer				
Shah-Altaf 2024	UK	surgeons that par_x0002_ticipated in the previous study	Hospital-based	Race Implicit Association Test.	Attitudes, Stereotype	ENT surgeons showed a slight implicit bias favoring White over Black people. While 42% acknowledged having unconscious racial bias, an equal proportion said they would change their behavior based on the results, and 85% found the Race IAT helpful for appraisal.
JimÃ©nezâ€Barbero 2024	Spain	professionals working in mental health units, hospitals and outpatient settings throughout Spain, belonging to the following professional groups: (a) psychiatry, (b) clinical psychology, (c) mental health nursing, (d) social work, (e) occupational therapy and (f) nursing assistant	Community, Hospital-based, Primary care	The Genderism and Transphobia Scale (GTS) , EANT [Trans Negative Attitudes Scale]); Ambivalent Sexism; e Spanish version of this scale Ferrando and Chico (2000)	attitudes	The study examined mental health professionals' attitudes toward trans individuals and their relation to sociodemographic factors. Males showed significantly higher levels of transphobia and gender bashing than females. Males also reported greater benevolent sexism, consistent with prior research linking sexism and transphobia. Older professionals and those with religious beliefs demonstrated more negative attitudes and higher sexism scores, though religious effects were weak. Transphobia was higher among psychiatrists, occupational therapists, and nursing assistants—possibly reflecting lower levels of training.
Alzahrani 2024	Canada	Ontario College of Pharmacists, pharmacists	Primary care	Implicit Associate Tests (IATs)	Attitudes, Stereotype	The study surveyed 407 community pharmacists, 56.1 % of whom were women with an average age of 46.9. Implicit Association Test (IAT) results showed a statistically significant moderate preference for white people over both Black (mean IAT = 0.41) and Arab people (mean IAT = 0.35). However, most pharmacists explicitly stated that they had no racial/ethnic preference, with 75.7 % expressing a neutral preference between Black and white and 66.6 % neutral between Arab and white. However, a slight preference for white individuals was observed. Demographic factors such as age, place of birth, race/ethnicity, and experience significantly impacted IAT scores. For example, older, Canadian-born, white pharmacists with

Reference (Author, Year)	Country	Population	Setting	Method for Assessing Bias	Construct	Main Finding Related to Bias
						more experience displayed higher implicit bias scores. A mild correlation was found between implicit and explicit bias, indicating as implicit bias increases, explicit bias tends to become more negative.
Van 2023	Netherlands	Healthcare professionals from seven different disciplines in the Netherlands.	Primary care, Hospital-based, Academic	No specific instrument or scale. Interviews and questionnaires.	Implicit Bias: Attitudes	The study found that Dutch healthcare professionals exhibit negative weight-related attitudes toward pediatric patients with obesity, with variations across different disciplines.
Dasgupta 2021	USA	335 participants, including healthcare workers and trainees from various disciplines.	Academic, Hospital-based	Disability Implicit Association Tests (IAT).	Attitudes and implicit biases.	The collected data reflects responses from 335 participants. Within this sample, there were both explicit and implicit biases towards individuals with physical disabilities. Prior to the IAT and educational module, when respondents were tasked with providing genetic testing recommendations, implicit biases and personal preferences for genetic testing and termination influenced respondents' clinical recommendations. Importantly, having previous professional experience with individuals with disabilities diminished biased clinical recommendations prior
Asante-Korang 2023	USA	Children waiting for pediatric heart transplantation in the United States.	Hospital-based	Implicit Association Test (IAT).<br>Análisis de datos clínicos Encuestas	attitudes and Stereotypes	Of 500 members, 18.2% completed the race IAT and 14% completed the SES IAT. Results showed moderate implicit racial bias favoring White individuals ($M = 0.33$, $d = 0.76$, $p < 0.001$) and strong SES bias favoring upper-class individuals ($M = 0.52$, $d = 1.53$, $p < 0.001$). Explicit race and wealth bias were weak, but explicit education bias was strong ($M = 5.22$, $d = 1.19$, $p < 0.001$). No significant correlations were found between implicit and explicit bias measures.
Barnard 2022	USA	Doctor of Pharmacy (PharmD) students from four universities in the Midwest and Southeast United States	Hospital-based, Academic	Health Care Provider HIV/AIDS Stigma Scale (HPASS)	Stigma Attitudes toward persons living with HIV/AIDS	The HPASS subscales demonstrated acceptable unidimensionality, good item and person separation reliability, and evidence of convergent validity. Poor functioning was observed in response categories 3 and 6 of the Likert scale, suggesting the need for category refinement. Two items (items 15 and 13 from Prejudice) were removed due to misfit.
Shawnika 2021	USA	Healthcare Professionals: Licensed physicians	Primary care	Instrument Used: Color Blind Racial Attitudes Scale (COBRA), which evaluates three subscales:	Attitudes	Providers with higher scores on the COBRA scale showed less willingness to discuss and prescribe PrEP to Black patients. Perceptions of patient adherence mediated the relationship between racial bias and willingness to prescribe PrEP.

Reference (Author, Year)	Country	Population	Setting	Method for Assessing Bias	Construct	Main Finding Related to Bias
		actively practicing in one of the 48 U.S. counties designated as high HIV incidence areas, with privileges to prescribe medications. Sample Size: 160 primary care providers.		Racial privilege ignorance. Institutional discrimination. Explicit racial issues.		Substance use history did not show a significant interaction with racial bias in the willingness to discuss or prescribe PrEP.
Hasret 2018	Turkey	The sample consisted of 729 students attending the departments of nursing, first and emergency aid, medical laboratory techniques, elderly care, and rehabilitation at Atatürk University in Turkey	Academic	The prejudice against obesity scale (GAMS-27)	Prejudice against obesity	The students' mean Prejudice Against Obesity Scale score was 75.54 ± 10.43, indicating a tendency toward prejudice. Male students' mean scores (78.20 ± 9.85) were significantly higher than those of female students (74.24 ± 10.46) ($p < .05$). A weak but significant positive correlation was found between prejudice scores and students' height, weight, and BMI ($p < .05$). No significant difference in prejudice scores was found according to department, grade, place of longest residence, physical activity level, or having overweight individuals in the family
Lakshminarayana 2018	USA	Healthcare providers (67 mental health nurses, 91 primary care nurses, 55 physicians, 62 psychiatrists, and 76 psychologists)	Primary care	Semantic Differential Scale (9 items): Measures negative Stereotypes using a 7-point Likert scale. Attribution Questionnaire (AQ-9) (9 items): Assesses emotional responses such as anger, pity, and fear on a 9-point Likert scale. Social Distance Scale (5 items): Measures the intention	Stereotypes discrimination	A significant positive correlation between Stereotypes and prejudice (β = 0.298, $P < .0001$). Prejudice significantly predicted social distancing (β = 0.190, $P = .002$). A significant indirect effect of Stereotypes on social distancing mediated by prejudice (β = 0.167, $P = .007$).

Reference (Author, Year)	Country	Population	Setting	Method for Assessing Bias	Construct	Main Finding Related to Bias
				to maintain social distance on a 4-point Likert scale.		
Hagiwara 2023	USA	general US population and subpopulations of physicians and non-physician healthcare workers.	Community	Race, Native American, Asian American and/or Arab-Muslim IATs	Attitudes, Stereotype	Among physicians, 8 percent were Black, 71.5 percent were White, 2.2 percent were Hispanic, and 17 percent were Asian. Fig. 1, the data show that both physicians and non-physician healthcare workers exhibited higher levels of implicit prejudice towards Black Americans and Arab-Muslim Americans compared to the general population. In contrast, physicians exhibit lower levels of implicit prejudice directed toward Asian Americans and Native Americans than the general population; other healthcare workers exhibited stronger implicit prejudice toward Asian Americans and Native Americans than the general population. Race IAT/explicit Anti-Black prejudice . On average, both physicians and non-physician healthcare workers in the sample initially exhibit significantly more anti-Black implicit prejudice compared to the general population (Table 2, Column 1). However, IAT score differences between physicians and the general population narrow and eventually disappear after controlling for demographic characteristics.
Quillin 2022	USA	Certified and trainees genetic counseling	Community	Race Implicit Association Test Implicit racial stereotyping was assessed with the medical cooperativeness IAT,	Stereotype Attitudes Cognition	Mean D-scores indicated moderate implicit bias linking Black faces with psychosis (M = 0.25), non-compliance (M = 0.21), and antipsychotic medication (M = 0.27). Around 38–39% of participants showed moderate-to-strong associations in this direction, while <9% showed such associations for White faces. D-scores across tasks were positively correlated (r = 0.27–0.41, p < 0.01). Higher training levels among psychiatric providers were linked to stronger bias in diagnostic and medication tasks, but not in compliance. Black and mixed-race participants had significantly lower odds of demonstrating strong bias compared to White participants; no differences were found for Hispanic or Asian respondents.

Reference (Author, Year)	Country	Population	Setting	Method for Assessing Bias	Construct	Main Finding Related to Bias
Bloch 2021	USA	medical students and psychiatric physicians in the United States	Community	Adapted Implicit Associations Tests (IATs)	Stereotype Attitudes Cognition	The mean D-score for the mood/psychosis IAT was 0.25 (SD= 0.38), the compliance/non-compliance IAT was 0.21 (SD= 0.39), and the antidepressant/antipsychotic IAT was 0.27 (SD= 0.33). A large proportion of the D-scores was observed to fall on the positive tail of the distribution (D-Scores ≥ .35) which denotes moderate-to-strong implicit associations pairing faces of Black individuals with psychosis, non-compliance, and antipsychotic words. Specifically, 39.12% (95% CI; 33.50–44.95%) of participants had moderate-to-strong implicit associations between Black faces and psychotic disorder words, 37.70% (95% CI; 31.69–44.00%) had moderate-to-strong implicit associations between Black faces and non-compliance words, and 38.72% (95% CI; 32.46–45.27%) had moderate-to-strong implicit associations between Black faces and antipsychotic medications. On the other hand, a small proportion of the D-scores was observed to fall on the negative tail of the distribution (D-Scores ≤ −0.35) which denotes moderate-to-strong pairing White faces with psychosis, non-compliance, and antipsychotic words. Specifically, 5.78% (95% CI ; 3.40–9.10%) of participants had moderate-to-strong implicit associations between White faces and psychotic disorder words, 8.33% (95% CI; 5.23–12.46%) had implicit associations between White faces and non-compliance words, and 2.98% (95% CI; 1.21–6.04%) had moderate-to-strong implicit associations between White faces and antipsychotic medications. Scores on the 3 tasks were positively and significantly correlated with each other, but the degree of correlation varied. Task 1 (Diagnosis) & Task 2 (Compliance) had a correlation of r=0.27 with a p-value <0.01. Task 1 (Diagnosis) & Task 3 (Medications) had a correlation

Reference (Author, Year)	Country	Population	Setting	Method for Assessing Bias	Construct	Main Finding Related to Bias
						of r=0.41 with a p-value <0.01. Lastly, Task 2 (Compliance) & Task 3 (Medications) had a correlation of r=0.32 with a p-value <0.01.<br><br>In terms of level of training, compared to medical students (reference group), psychiatric physicians with higher levels of training had significantly stronger associations pairing Black faces with psychotic disorders or conversely White faces with mood disorders (psychiatric resident βΔD = 0.20, p < 0.001, psychiatric fellow βΔD = 0.23, p < 0.01, board-certified psychiatrist βΔD = 0.21, p < 0.05). These results were replicated in the antidepressant/antipsychotic IAT with the exception of board-certified psychiatrists (psychiatric resident βΔD = 0.16, p < 0.01, psychiatric fellow βΔD = 0.19, p < 0.01, board-certified psychiatrist βΔD = 0.04, p = NS). In the compliance/non-compliance IAT, associations with training level were smaller in magnitude and were not statistically significant (psychiatric resident βΔD = 0.07, p = NS, psychiatric fellow βΔD = 0.06, p = NS, board-certified psychiatrist βΔD = 0.01, p = NS)<br><br>or the positive D-score tail (i.e. providers pairing Black faces with psychosis/non-compliance/antipsychotics), compared to White participants, Black participants had significantly lower odds of having moderate-to-strong associations (mood/psychosis IAT: OR = 0.23 p < 0.01, compliance/non-compliance IAT: OR = 0.30 p < 0.01, antidepressant/antipsychotic IAT: OR = 0.20, p < 0.01). Furthermore, compared to White participants, those in the race/ethnicity category “Native American, Mixed or other” also had significantly lower odds of having moderate-to-strong associations pairing Black faces with psychotic disorders/non-compliance/antipsychotic words (mood/psychosis IAT: OR = 0.23, p = 0.05, compliance/non-compliance IAT: OR = 0.26, p < 0.05, antidepressant/antipsychotic IAT: OR = 0.21, p < 0.05). For Hispanic and Asian participants, the odds for moderate-to-strong associations were not significantly different to those of White participants across tasks.
Dos 2017	Brazil	All 915 dental students attending those schools were invited to complete the survey. Participation was	Primary care, Academic	The survey on students’ perceptions of and attitudes toward poverty consisted of 32 items derived from studies by Atherton and Gemmel19 and Gilens.20	attitudes toward people living in poverty and the extent to which their perceptions were	In a survey of 766 Brazilian dental students (83.7% response rate), participants showed slightly positive attitudes toward people living in poverty, though 35% viewed them as different from the general population. Most expressed willingness to treat underserved patients, which was significantly associated with beliefs about poverty (OR = 1.65; 95% CI: 1.41–1.94). While students held altruistic intentions, the study suggests a limited understanding of poverty may hinder their ability to act on them.

Reference (Author, Year)	Country	Population	Setting	Method for Assessing Bias	Construct	Main Finding Related to Bias
		voluntary and anonymous. Participants signed an informed consent form. Dental schools in Brazil are divided into public and private institutions.			associated with their willingness to treat those patients.	
Johnson 2017	USA	f resident physicians working in a pediatric ED	Academic	We used the Adult and Child Race IATs to assess implicit bias.	Attitudes	According to standard interpretations of IAT scores,24 we found moderate pro-white/antiblack bias on both the Adult Race (M=0.49, SD=0.34) and Child Race IAT (M=0.55, SD=0.37). There was no significant difference between Adult and Child Race IAT scores (difference=0.06, p=0.15). As shown in Figure 3, most residents had IAT scores consistent with pro-white/anti-black racial bias on both the Adult Race IAT (85%) and Child Race IAT (91%). In linear models adjusting for practice effect, none of the resident characteristics (i.e., specialty, gender, race, age, and training year) were associated with Adult or Child Race IAT scores
JM 2016	Spain	residents	Hospital-based	Discrimination questionnaire (EVE-D)	Attitudes	Over 60% of participants agreed with the first 20 discrimination scenarios, all statistically significant. Items 26 and 27 had lower agreement (38% and 36%) but remained significant. Only 11% agreed with a control question suggesting economic justification for limiting care by age, indicating low acquiescence. Factor analysis revealed three components explaining 37.06% of variance: (1) direct exclusion from high-cost programs, (2) indirect discrimination through delays or unequal treatment, and (3) differential treatment expressed as overprotection or paternalism toward older adults.
Blair 2014	USA	Adult primary care clinicians in the Denver metropolitan area were recruited in a prior study on ethnic/racial attitudes	Hospital-based	Two Implicit Association Tests (IATs) to measure implicit ethnic and racial biases. words require another response, compared with the reverse pairing.	Attitudes	Black patients received equivalent treat- ment intensification, but had lower medication adher- ence and worse hypertension control than white patients; Latino patients received equivalent treatment intensification and had similar hypertension control, but lower medication adherence than white patients. Differences in treatment intensification, medication adherence and hypertension control were unrelated to clinician implicit bias for black patients (P=0.85, P= 0.06 and P=0.31, respectively) and for Latino patients (P=0.55, P=0.40 and P=0.79, respectively). An increase in clinician bias from average to strong was associated with a relative change of less than 5 % in all outcomes for black and Latino patients.

Reference (Author, Year)	Country	Population	Setting	Method for Assessing Bias	Construct	Main Finding Related to Bias
Blair 2013	USA	Experienced primary care providers and Community members	Primary care	Implicit bias. The IAT measures the strength with which concepts (e.g., African American and White people) are associated with attributes (e.g., good and bad	Attitudes	With a 60% participation rate, the PCPs demonstrated substantial implicit bias against both Latinos and African Americans, but this was no different from CMs. Explicit bias was largely absent in both groups. Adjustment for background characteristics showed the PCPs had slightly weaker ethnic/racial bias than CMs (Community members)
Waller 2012	USA	Nursing and Psychology majors	Primary care, Community	The IAT was used to measure participants' implicit attitudes towards overweight individuals.	Attitudes	A statistically significant implicit bias towards overweight individuals was detected in both subject groups and in both target settings (medical vs. non-medical). Stronger weight bias was found when the stimulus targets were female than male.
Maina 2018	USA	A comprehensive literature search of several databases between May 2015 and September 2016 identified 37 qualifying studies.	Primary care	IAT	Attitudes	This systematic review included 37 studies on implicit bias in healthcare. Most (31) reported pro-White or anti-minority bias among healthcare providers. Fourteen studies assessed its link to clinical decision-making; 6 found associations between higher bias and disparities in treatment, empathy, and pain management. All 7 studies examining real-world patient–provider interaction found that stronger bias was linked to poorer communication. Only 2 studies examined real-world health outcomes; 1 found an effect. Of 2 intervention studies, just 1 reported a reduction in bias. Findings highlight the need for more real-world studies and effective strategies to mitigate bias in healthcare delivery.
Fraiman 2023	USA	neonatal intensive care units Staff	Hospital-based	Our survey included three parts: 1) Demographics, 2) Assessing equity and the impact of bias and, 3) "How are we doing?" All survey questions were optional. Survey was initially developed by an investigator and underwent iterative revisions until consensus was reached by the study team, which included physician and nursing leadership, attending physicians, clinical fellows and bedside nursing staff	Attitudes	We received 178 responses. More respondents agreed that bias had a greater impact on others' vs. their own behaviors (KWT $p<0.05$). Respondents agreed that behaviors were influenced more by implicit than explicit biases (KWT $p<0.05$). Qualitative analysis resulted in nine unique themes. Themes were then grouped into four major domains: 1) the impact of bias, 2) causes of bias, 3) strategies to mitigate bias, and 4) factors to consider when creating interventions (Table 3).

Reference (Author, Year)	Country	Population	Setting	Method for Assessing Bias	Construct	Main Finding Related to Bias
Hagiwara 2020	USA	Providers in health context of implicit prejudice and Stereotypes (provider to patient communication)	Primary care	IAT	Attitudes	Researchers are encouraged to use multiple implicit measures to further investigate how, why, and under what circumstances providers' implicit bias predicts provider-to-patient communication and treatment recommendations. Such efforts will contribute to the advancement of both basic social psychology/social cognition research and applied health disparities research: a better understanding of implicit social cognition and a more comprehensive identification of the sources of widespread racial/ethnic healthcare disparities, respectively.
Jones 2021	Canada	27 phy_x0002_siotherapists who attended the seminar. The second cohort consisted of physiotherapists who did not attend the semi_x0002_nar, were licensed in the province of Alberta and were invited to complete an online survey	Primary care	(The Attitude Towards Obese Persons, ATOP; (Beliefs About Obese Persons, BAOP	Attitudes, Stereotype	Physiotherapists attending a one-day seminar on bariatric care showed modest improvement in beliefs about obesity, with BAOP scores increasing from 17.4 to 22.3 ($p = .001$). This suggests greater acknowledgment of obesity's multifactorial causes. However, no significant differences in attitudes (ATOP scores) were found by gender, experience, or clientele. Compared to an online group (mean BAOP = 19.4), the seminar group still demonstrated implicit weight stigma, potentially reinforced by the seminar's focus on practical challenges. This highlights that educational efforts may unintentionally increase stigma if focused too heavily on logistical difficulties rather than bias awareness.
McQuad 2021	USA	practicing pharmacists	Hospital-based	Implicit Attitudes Test (IAT) and explicit attitude questionnaire	Attitudes, Stereotype	Forty-two studies met inclusion criteria. Seventeen used implicit measures (15 IAT, 2 subliminal priming), and 25 used vignette-based designs to examine the influence of patient characteristics. Racial/ethnic bias was most common ($n = 27$), followed by gender, age, and weight. Implicit bias was found in 35 studies, often linked to poorer clinical decision-making. Biases affected diagnoses, treatment, and provider–patient interactions. Sociodemographic factors (e.g., provider gender, race, country of training) influenced bias levels. Implicit bias did not always align with explicit attitudes, reinforcing the need for indirect assessment tools.

Reference (Author, Year)	Country	Population	Setting	Method for Assessing Bias	Construct	Main Finding Related to Bias
Dahlman 2024	Sweden	Nurses and physicians	Hospital-based	IAT<br>feeling thermometer self-assessments (for explicit bias)	Attitudes	Originally developed and validated (coefficient alpha = .87; test–retest reliability = .84)<br>The median MCRS score was 44 for both patients with drug use and patients in OAT, and 51 for patients with depression. Scores regarding patients with drug use and OAT patients displayed similar minimum, maximum and interquartile range values as well, while the scores regarding patients with depression displayed a higher minimum and much smaller interquartile range.<br><br>A greater share of the respondents would prefer not to work with patients in active drug use, who perceived them as irritating or particularly difficult to work with, while a slightly lower share agreed that they felt satisfaction working with them. In addition, slightly fewer indicated that they could usually help drug-using patients feel better or that they would mind working inconvenient hours to care for them.<br><br>We found no significant associations between the MCRS scores for either drug use or OAT, and age, sex, profession or duration of professional experience
Tzavella 2024	Greek	Nursing staff	Primary care, Hospital-based	Questionary	Attitudes and beliefs	From the analysis of the data, it was found that the majority of the participants were female (76.7%).<br>Regarding the results of the participants' scores on the 3 dimensions of the discrimination behavior scale, the highest average value was recorded for the dimension of communication (mean=5.85), and the lowest for that of discrimination ((mean=3.03). Cronbach's reliability coefficients were above the acceptable limit, in all dimensions, indicating acceptable reliability. With reference to the results of the participants' scores on the 3 dimensions of the discrimination behavior scale, the score on the "Communication" dimension ranged from 4.11 to 6.67 points, with the mean value being 5.85 points (SD=0.59 points). Correspondingly, the score<br>on the dimension "Difficulties" ranged from 1.38 to 5.25 points, with the mean value being 3.35 points (SD= 0.92 points), while for the dimension "Discriminations" it ranged from 1.00 to 5.67 points, with the mean value being 3.03 points (SD=1.12 points).
Sabin 2022	USA	Clinical and Medicine students / Residents	Academic, Hospital-based	a bias awareness measure developed by<br>Girod et al. [11] to assess pre–post-bias awareness	Attitudes and cognition (Bias Awareness)	

Reference (Author, Year)	Country	Population	Setting	Method for Assessing Bias	Construct	Main Finding Related to Bias
				change among academic physicians following an implicit bias educational intervention on gender bias in faculty advancement for hiring committees		
Gorade 2023	India	medical graduates (undergraduates and postgraduates)	Academic	An online cross-sectional survey:<br><br>2. Attitudes, Beliefs, and Practices towards LGBTQ: The California State University, Northridge (CSUN) questionnaire and had 14 questions.<br>3. attitudes toward LGBTQ people: Homosexuality Attitude Scale (HAS) using a 7-item questionnaire<br>4. perceptions of homosexuals: Acceptance of Homosexuals (AOH) by the Hong Kong Home Affairs Bureau.	Attitudes Stereotype Cognition	The attitude of medical students toward LGBTQ Around 87.6% (n = 263) of participants reported to be<br>comfortable having a friend with a homosexual orientation in their circle and 63% (n = 189) disagree that acceptance of LGBTQ people has anything to do with the morality of our society. Positive attitudes were reflected in 84% (n = 252) of participants, who believed that homosexuality is not a mental illness and around 12% believed that it is a mental illness, with 4% refraining from answering. Of all the participants, 72.6% (n = 218) had an optimistic attitude toward LGBTQ people when it comes to interacting and living with them. On sexual orientation, 60.3% (n = 181) believe that the love shared among both individuals from the same sex is no different from that between the opposite sex, and a similar proportion of participants (60.6%, n = 182) feel that homosexuals are not majorly involved in deviant sexual behavior such as child<br>molestation, rape, voyeurism, and so on [Figure 1].<br>Acceptance of LGBTQ people. According to the AOH scale, while evaluating acceptance level, 88.3% (n = 265) of the students showed acceptance of an LGBTQ person as a friend, 90.6% (n = 272) as a neighbor, 91.3% (n = 274) as a teacher, and 86% as any one of their family members. Around 91.6% (n = 275) of participants are comfortable working with people having a homosexual orientation [
FitzGerald 2017	Switzerland	Physicians, Nurses, 'Clinicians', or 'mental health professionals' (at least some of whom were nurses and physicians), Psychologists, Medical Students	Academic, Primary care	Implicit Association Test (IAT) and Assumption Method	Stereotype	A review of 42 studies found consistent evidence of implicit bias among healthcare professionals, especially regarding race, gender, age, weight, and socioeconomic status. These biases were linked to lower quality of care, affecting diagnoses, treatment decisions, and patient interactions. Sociodemographic factors of providers influenced bias levels. Implicit and explicit attitudes often differed, limiting the reliability of self-report measures.

Reference (Author, Year)	Country	Population	Setting	Method for Assessing Bias	Construct	Main Finding Related to Bias
Pool 2023	USA	Clinical team	Primary care, Hospital-based	Groot Critically Reflective Diagnosis Protocol (DCRDP) can be used as a data analysis tool to evaluate group dynamics as an essential foundation for exploring how interactions can bias collective clinical decision-making	Stereotypes attitudes	The DCRDP was revealed as a practical tool for examining group decision-making bias. It can be adapted to a variety of clinical, educational, and other professional settings as an impetus for recognizing the presence of team-based bias, engaging in reflexivity, informing the design and testing of implementation strategies, and monitoring long-term outcomes to promote more equitable decision-making processes in healthcare
Abbott 2023	UK	Obesity specialist HCPs	Primary care	the BiasProof mobile device test, based on the Implicit Association Test	Stereotypes and attitudes	82 of the 113 HCPs who attended the webinar consented to contribute data to the study. Over half (51%) had an implicit weight bias against PLwO. Most (90%) agreed/strongly agreed that obesity services are too weight focused and that patients should not be weighed at every appointment (86%). Perceived benefits of taking a non-weight focused approach included patient-led care, reducing stigma and supporting patient wellbeing, while perceived barriers included loss of objectivity, inducing risk and difficulty demonstrating effectiveness. Our findings indicate that half of obesity specialists HCPs in our sample of 82 providers, who are primarily dieticians and psychologists, have an implicit weight bias against PLwO. HCPs feel that a weight-focused approach within services was a barrier to patient care, but that there is a lack of alternative non-weight focused measures. Further research is needed into substitute outcome measures for clinical practice, also seeking the views of PLwO, and into interventions to address implicit weight bias.
Thirsk 2022	Canada	Nurses included registered nurses (RN), practical nurses, psychiatric nurses, and nurse practitioners (NP)	Academic, Primary care, Community	survey, vignette, interview	Stereotype, cognition	Mill et al. (2013) found that nurses reported stigmatizing behaviors toward HIV-positive patients, including room isolation, lack of basic care, and delayed services. Boyle et al. (2019) observed that health students showed weight and racial bias in pediatric pain scenarios, attributing pain in higher-weight children to psychological causes and perceiving African-American children as experiencing more pain. Students with lower weight bias showed more sympathy and willingness to help.

Reference (Author, Year)	Country	Population	Setting	Method for Assessing Bias	Construct	Main Finding Related to Bias
Dovidio 2019	USA	US medical students	Academic	A clinical vignette-based online survey with randomized patient race (Black or White).<br><br>The Modern Racism Scale to assess explicit racism.<br><br>Implicit Association Tests (IATs) to measure two forms of implicit racism.<br><br>A standard set of verbal stimuli to assess implicit racial prejudice.	Cognition	No significant differences in explicit racism, implicit racial prejudice, or heterosexism were observed by demographic or educational variables. Despite the vignette describing a monogamous patient, 90% of participants anticipated risk compensation (e.g., increased condomless sex), and 30% anticipated new sexual partners. Only 47% believed the patient would be highly adherent to PrEP. Nonetheless, 77% indicated they would probably or definitely prescribe PrEP.
Turmo 2023	Nicaragua	Health-care students	Academic	N-GAMS de 33 items	Attitudes Stereotype	The initial Cronbach's alpha was satisfactory (0.747), with acceptable reliability and item-total correlations across the 12 items. After removing items v29, v30 (biomedical focus), and v43 (professional expectations) due to low item-total correlations (< 0.2), the revised Cronbach's alpha improved to 0.809. All factor loadings exceeded 0.4, and model fit indices were acceptable: CFI = 0.917, TLI = 0.899, RMSEA = 0.08, and SRMR = 0.06.

Reference (Author, Year)	Country	Population	Setting	Method for Assessing Bias	Construct	Main Finding Related to Bias
McGhie-Fraser 2023	Netherlands	Healthcare professionals working with Patients with persistent somatic symptoms (PSS)	Academic, Primary care, Community	HC-PAIRS (Health Care Provider Pain and Impairment Relationship Scale), B-IPQ (Brief Illness Perception Questionnaire), PQ-Phys (Physician Questionnaire), CFS-KAB (Chronic Fatigue Syndrome Knowledge and Attitudes Questionnaire), MCSS (Modified Condition Attribution Scale), IPQ-R (Illness Perception Questionnaire-Revised), MUS-Q (Medically Unexplained Symptoms Questionnaire), CFS-AS (Chronic Fatigue Syndrome Attitude Scale), CAT (CFS Attitude Test), CPSS-HCP (Chronic Pain Stigma Scale for Health Care Providers), ODPQ (Obesity and Disease Perceptions Questionnaire), GP Attitudes to CFS (General Practitioner Attitudes to Chronic Fatigue Syndrome), PDPQ (Patient-Doctor Relationship Questionnaire), PABS (Pain Attitudes and Beliefs Scale), FAS (Fibromyalgia Attitude Scale), IBIS (Irritable Bowel Syndrome Instrument), FND-SS (Functional Neurological Disorder Stigma Scale), KAB-Pain (Knowledge and Attitudes About Pain), PCP-PSQ (Primary Care Provider Pain Stigma Questionnaire), PAS (Pain Attitude Scale),	Stereotype, cognition	Ninety studies met inclusion criteria, using 62 different questionnaire-based instruments. Stereotypes were assessed in 92% of tools, prejudices in 52%, and discrimination in 19%. Instrument development quality was generally rated as doubtful. Commonly assessed psychometric properties included construct validity, structural validity, internal consistency, and reliability. Evidence on content validity was inconsistent or unclear.

Reference (Author, Year)	Country	Population	Setting	Method for Assessing Bias	Construct	Main Finding Related to Bias
				SESAMS (Self-Efficacy in Managing Somatic Symptoms), WAP-FM (Willingness to Accept Patients with Fibromyalgia)		

Reference (Author, Year)	Country	Population	Setting	Method for Assessing Bias	Construct	Main Finding Related to Bias
Featherston 2020	Australia	Health professionals	Primary care, Hospital-based, Community, Academic	clinical vignettes or case analogues (either written, audio or videotaped)	Stereotype, cognition	Decision types were categorized into assessment (n = 86), diagnosis (n = 68), treatment (n = 38), prognosis (n = 34), child placement (n = 6), school placement (n = 4), and genetic likelihood (n = 1). Most studies (n = 82) evaluated one decision type; the rest assessed multiple types. Biases were commonly related to stereotyping based on gender (n = 40), race/ethnicity (n = 35), class (n = 26), and age (n = 8), among others. Cognitive biases included confirmation (n = 12), anchoring (n = 11), diagnostic overshadowing (n = 5), and labeling (n = 5).
Crump 2025	USA	Pre medical, medical student and medical graduated	Academic	10-hour LGBTQ health curriculum, Structural Foundations of Health Survey, structured in-depth interviews, survey design, pre/post questionnaire, skills and knowledge-based interventions, 1-hour multimedia interactive lecture, Implicit Relational Assessment Procedure (IRAP), high-fidelity simulation, web-based survey, pretest-posttest design, ethics education with IAT, health equity curriculum, seminars and patient assessments, focus groups, self-examination prompts, determinants of health course, academic workshop, student-led initiatives, course interventions, antiracism curriculum, open-ended written questionnaire, three-part implicit bias training, skills-based curriculum, obesity education programs, semi-structured interviews, Likert-scale surveys, workshops, facilitated discussions, verbal	Attitudes, Stereotype	Implicit bias was least studied at the pre-medical level. Among medical and graduate students, findings showed preference for male, white, non-LGBTQIA+, and thin patients, suggesting current mitigation tools are insufficient and may reinforce health disparities. Implicit bias may contribute to confirmation or anchoring bias, leading to misapplied frameworks like social determinants. This study uniquely examined implicit bias across educational stages, highlighting opportunities for early intervention. However, limitations include exclusion of studies before 2017, reliance on published literature (potential publication bias), and inability to assess causality or the influence of measurement variability across studies.

Reference (Author, Year)	Country	Population	Setting	Method for Assessing Bias	Construct	Main Finding Related to Bias
				responses, written evaluations, resident applications, and templated spreadsheets.		
Derbyshire 2024	UK	nurses (n= 24,545), (2) other healthcare providers (n= 57,818) and (3) non-healthcare providers (n= 547,966)	Primary care, Hospital-based, Community	Implicit Association Test (IAT).	Attitudes, Stereotype	Most participants reported no explicit bias toward people with disabilities (PWD), with HCP-Nurses (69.6%) and HCP-Non-Nurses (66.4%) scoring more positively than non-HCPs (64.7%). Explicit bias scores were significantly more negative among non-HCPs ($p < .001$), though effect sizes were small. Conversely, implicit bias (IAT D-scores) was higher among HCP-Nurses (45.2%) than HCP-Non-Nurses (43.1%) and non-HCPs (41.1%), showing a reversal: those with the most positive explicit attitudes exhibited the highest implicit bias. This pattern was statistically significant but with small effect sizes. Over a 15-year span, implicit attitudes remained stable across groups, with no evidence of improvement.
McGhie-Fraser 2024	UK	Healthcare Professionals	Primary care, Hospital-based	Persistent Somatic Symptom Stigma scale for Healthcare Professionals (PSSS-HCP)	Attitudes, Stereotype	Following expert consensus, 40 items were retained for cognitive interviews. After Round 1 (n = 11), 20 items were removed, 3 added, and 5 amended. After Round 2 (n = 7), 4 items were removed and 3 amended. No major issues with relevance, clarity, or social desirability were found. The final PSSS-HCP scale includes 19 items across 3 domains (stereotype, prejudice, discrimination), scored 19–95; lower scores indicate less stigma. Items 1, 5, 6, 7, 15, and 16 are reverse-scored. Developed in a UK healthcare setting, the scale requires further validity and reliability testing. Structural factors—e.g., lack of policy, guidance, or senior support—were found to influence healthcare professionals' responses and perceived stigma.
Lasalvia 2024	Italy	Ambulance personnel of the non-profit private ambulance service organization	Hospital-based	15-item OMS-HC	Attitudes, Stereotype	The original factor structure of the OMS-HC, comprising three subscales, was confirmed. The internal consistency for the OMS-HC total score was good (α=0.75) and acceptable for the subscales (Social Distance α=0.66; Attitudes α=0.59; Disclosure/Help-Seeking α=0.61). One-third of respondents displayed stigmatizing attitudes on half of the OMS-HC items. Higher scores were associated with being male, having lower levels of

Reference (Author, Year)	Country	Population	Setting	Method for Assessing Bias	Construct	Main Finding Related to Bias
						education, and working as both rescuers and ambulance drivers and with feeling uncomfortable when dealing with patients with mental illness. Overall, stigmatizing attitudes towards individuals with mental illness are prevalent among ambulance staff. The Italian version of the OMS-HC for ambulance personnel demonstrated satisfactory psychometric properties and is recommended for evaluating training programs targeting this population.
Brent 2024	USA	Orthopaedic surgeons (Residents, faculty, and fellows)	Hospital-based	Race IAT<br>gender-career association test	Stereotype	The mean IAT D-score for race was 0.29 6 0.4, indicating a slight bias toward associating "White" with "good." However, D-scores demonstrated a wide range, as can be seen in the distribution curve (Fig. 1), showing a spectrum of preferences for Black or White, and 23% of the respondents (96 of 419) had scores indicating little to no preference. Furthermore, 27% of respondents (112 of 419) had D-scores $ 0.65, corresponding with a strong preference for "White" and "good."<br>The results of the gender IAT showed a slight bias toward associating women with family and home and associating men with work and career, with a mean D-score of 0.24 6 0.3 (Fig. 2). A total of 31% (129 of 419) of respondents had little to no preference (D-score > -0.15 and < 0.15) and 14% (60 of 419) strongly associated women with family and home and men with work and career (D-score >0.65).
Stef 2023	Saudi Arabia	Students	Academic	online questionnaire:<br>(1) a scale for social distancing<br>(2) a scale about negative attitudes and stereotyping toward diabetes and those diagnosed with diabetes	attitudes<br>Stereotype	The range of standardized loadings in the negative attitude and serotyping factor was −0.22 to 0.98. The result of CFA showed that the model had a moderately acceptable fit. The goodness of model fit parameters were: CFI = 0.911, TLI = 0.885, RMSEA = 0.136, and SRMR = 0.049 (Table 4). The range of<br>standardized loadings in the social distancing factor was 0.12 to 0.70. The result of CFA showed that the model had a moderately acceptable fit. The goodness of model fit parameters were:<br>CFI = 0.85, TLI = 0.79, RMSEA = 0.111, and SRMR = 0.067 (Table 5).<br>The Cronbach α coefficient for the social distance and negative attitudes and stereotyping scales were 0.94 and 0.75, respectively.
Gandhi 2015	India	undergraduate medical students and interns	Academic	1. The Sex Education and Knowledge about Homosexuality Questionnaire (SEKHQ)	Attitudes<br>Cognition	Females showed more positive attitudes toward homosexuality than males, though knowledge levels did not differ significantly. Interns and third-year students had higher knowledge scores than early-year students (p = 0.046), but this was not reflected in attitudes. Non-Hindus had greater knowledge than Hindus, but attitude differences were not statistically

Reference (Author, Year)	Country	Population	Setting	Method for Assessing Bias	Construct	Main Finding Related to Bias
				2. The Attitudes towards Homosexuals Questionnaire (AHQ)		significant. Knowledge correlated positively with age and negatively with prejudice. In regression analysis, knowledge and gender predicted attitudes (explaining 16.3% of variance); religion predicted knowledge (3.2%). Both models were statistically significant ($p < 0.001$).
Adel 2017	Canada	1000 registered nurses across the province of British Columbia, and only 170 nurses completed and returned the instrument.	Hospital-based	A critical cultural competence (CCC) scale was used to measure the registered nurses' perceptions of critical cultural competence in a multicultural context. The Critical Cultural Competence scale comprised 43 items.	N/A.	
Gleicher 2022	USA	Residents/fellows, Attendings, Physicians: unspecified, Mixed health professionals, Nurses, social workers, and other members of the health care system, Medical students	Hospital-based, Academic	Implicit Association Test (IAT), Perspective-Taking Intervention, High-Fidelity Simulation Intervention, Web-Based Decision Support Too, Cultural Competency Education Session, Experimental Vignettes with Randomized Patient Characteristics, Survey and Recall Methods.	Stereotype	
Brown 2017	USA	Undergraduate nursing students (N = 265	Academic	Online survey assessing transprejudice attitudes, openness to experience, attribution of cause for being transgender, and empathic concern SOLO NOMBRE DEL INSTRUMENTO	Attitudes	Confidence in providing culturally competent and affirming health care to diverse populations was associated with having received educational information on transgender issues and personally knowing a transgender individual. A significant positive correlation was found between beliefs that being transgender is due to genetics and holding more accepting attitudes toward transgender individuals.
Evridiki 2016	Greece	Healthcare professionals (physicians, nurses, administrative staff and	Hospital-based	Opinions about Mental Illness scale (OMI-G), Social Distance Scale (SDS), and Level of Contact Report (LCR).	Attitudes	Higher educational level and more frequent contact with people with mental illness were associated with less stigmatizing attitudes. Nurses demonstrated significantly higher levels of stigma compared to physicians. Contact was a significant predictor of more positive attitudes.

Reference (Author, Year)	Country	Population	Setting	Method for Assessing Bias	Construct	Main Finding Related to Bias
		auxiliary personnel) working in five general hospitals in Greece (N=479).				
Gena 2019	USA	Preclinical and clinical chiropractic students (N=450 invited, 143 participated) and faculty (N=46 invited, 30 participated).	Academic	Beliefs About Obese Persons (BAOP) scale and Attitudes Toward Obese Persons (ATOP) scale.	Attitudes	Both students and faculty demonstrated negative attitudes toward obese persons.<br>Clinical students had more positive attitudes than preclinical faculty on the ATOP scale.<br>BAOP scores suggested more belief in biological causality among students than faculty.<br>The regression analysis did not show significant differences between groups except for clinical students vs preclinical faculty.
Diana 2017	United States	White medical students (N=2394) graduating in 2014 from 49 U.S. medical schools.	Academic	No formal standardized instrument for implicit bias measurement; measures included perceived learning orientation (3-item scale), hours of training about racial disparities, and self-assessed preparedness (3 scales: self-efficacy, skills, and interest)	Learning orientation regarding interracial interactions, preparedness to care for minority patients (including self-efficacy, skills, and interest	White students' perceptions of their medical schools' learning orientation regarding interracial interactions (β = 0.18, P < .001) and their own learning orientation (β = 0.38, P < .001) were positively associated with preparedness. Learning orientation moderated the effect of training about disparities on preparedness (interaction β = 0.10, P < .01)
Daisy 2020	United Kingdom	Breast cancer healthcare professionals (e.g. surgeons, radiation oncologists, medical oncologists, general practitioners, nurses, residents).	Hospital-based	The included studies used a variety of tools: Implicit Association Test (IAT), questionnaires, case vignettes, and semi-structured interviews	Attitudes	In 14 of the 20 studies included, there was evidence suggesting that age influenced treatment recommendations, even when clinical profiles were equivalent. Healthcare professionals more frequently recommended less aggressive treatment for older patients. Implicit and explicit biases were reported to influence decision-making, with age often used as a heuristic

Reference (Author, Year)	Country	Population	Setting	Method for Assessing Bias	Construct	Main Finding Related to Bias
Mary 2023	USA	physicians and physicians-in-training (including fellows, residents, and medical students) in North America	Primary care	Implicit Association Test (IAT) – adapted<br>Crandall Anti-fat Attitudes Questionnaire – adapted<br>Professional Weight Bias Scale – developed by investigators	Stereotype	Physicians showed strong implicit preference for nonobese physicians (Cohen's d = 1.13), high levels of explicit WB, and moderate levels of professional WB. Positive correlations were found among all three types of bias (r = 0.38–0.41, p < 0.001).
Sabin 2009	USA	Medical Doctors	Primary care, Community	Race Attitude Implicit Association Test (IAT)	Attitudes	MDs' implicit and explicit attitudes about race follow the same general pattern seen in the public samples; the majority held implicit preferences for Whites over Blacks
Alexander 2007	USA	Internal medicine and emergency medicine residents at four academic medical centers	Hospital-based	Implicit Association Tests (IATs	Stereotypes	As physicians' prowhite implicit bias increased, so did their likelihood of treating white patients and not treating black patients with thrombolysis (P=.009)
Ahn 2022	USA (Hawaii)	49 practicing OB-GYNs	Primary care, Academic	Implicit Racial Attitudes and Associations Among Obstetricians in Hawai'i: a Pilot Study.	attitudes	Overall, participants demonstrated an implicit preference for White faces compared with NHOPI (d = 0.62), Asian American (d = 0.48), and Black (d = 0.59) faces.
Emily 2021	United States	Five departmental/divisional grand rounds across three different academic medical centers in New York, USA	Academic	Twelve-item retrospective pre-intervention/post-intervention survey.	Comfort and confidence in recognizing and managing implicit bias, debriefing with learners, and role-modeling behaviors.	We received 116 completed surveys from 203 participants (57% response rate). Participants' self-reported increased comfort and confidence in recognizing and managing bias. Qualitative analysis revealed themes such as speaking up, reflecting on biases, and engaging learners
Papish 2019	Canada	Undergraduate students (n = 382), medical students (n = 118), and	Academic	Opening Minds Scale for Health Care Providers (OMS-HC), Implicit Association Test (IAT)	Explicit stigma, implicit stigma, disclosure/help-seeking attitudes	Psychiatrists had significantly lower explicit and implicit stigma than students. Medical students had better explicit attitudes than undergraduates. Higher stigma was associated with lower diagnosis rates. OMS-HC and IAT scores were not significantly correlated. Structured contact-based education may reduce explicit stigma, but implicit attitudes are harder to change

Reference (Author, Year)	Country	Population	Setting	Method for Assessing Bias	Construct	Main Finding Related to Bias
		psychiatrists (n = 22)				
Evers 2023	Netherlands	Ninety-nine GPs participated in this randomized online study	Primary care	Implicit Association Tasks (IATs) were used to measure two IGBs, related to lifestyle and communication.	Implicit gender biases (IGBs)" and associated cognitive associations about lifestyle and communication.	Female GPs exhibited a significant lifestyle IGB ($p < 0.001$). GPs of both genders exhibited a significant communication IGB ($p < 0.001$). Male patients were more likely to be recommended medication treatment, while female patients were more likely to be assigned lifestyle interventions. IGBs predicted these differences in treatment decisions
Puhl 2014	USA	680 participants (329 registered nurses and 351 Nursing students)	Academic	IAT;<br>Anti-fat Attitudes questionnaire(AFA)	implicit and explicit antifat bias;<br>Stereotypes about individuals	Both nursing students and registered nurses demonstrated implicit and explicit antifat bias; explicit bias was significantly higher among students, while implicit bias scores were for both groups.
Phelan 2017	Usa	4732 first-year medical students	Academic	Implicit Association Test (IAT); Feeling Thermometer (explicit attitudes measure)	Implicit bias, explicit bias, attitudes toward gay and lesbian individuals	Among heterosexual students, mean implicit bias decreased slightly (Cohen's $d = 0.11$), while explicit bias decreased more substantially ($d = 0.64$). Bias decreased more among students reporting a more positive diversity climate, more contact with sexual minorities, and more training. Role modeling had inconsistent effects.
Schatz 2022	USA	patients with cancer (N=909) including oversampling) caregivers and practicing oncologist (N=208)	Primary care, Hospital-based	Custom national survey instrument developed by ACS CAN, NCCN, NMQF, and Public Opinion Strategies.	Attitudes, perceived discrimination, implicit and explicit bias	AA/B or H/L patients were substantially more likely to report that the healthcare system treats people unfairly based on their racial or ethnic background... Additionally, AA/B and H/L patients and caregivers were also more likely to report that they felt their care team made overall assumptions about them... or more precisely, assumptions about their financial circumstances.<br>A major finding was that 56% of oncologists reported that it was not possible they ever had unintentional bias in their care delivery for their patients.<br>A majority of oncologists (62%) agreed that non-White patients are more likely to receive poor quality care than W patients

Reference (Author, Year)	Country	Population	Setting	Method for Assessing Bias	Construct	Main Finding Related to Bias
Almutairi 2017	Canada	registered nurses who are currently working in various hospitals in the province of British Columbia	Hospital-based	critical cultural competence (CCC) scale;	Attitudes, Stereotype	A total of 1,000 nurses were invited; 170 responded (17%). Most were female (90.6%), aged 23–67 (M = 43.7), and held a bachelor's degree in nursing (59%). Staff nurses represented 88.7% of the sample. Average professional experience was 16.6 years. Only 38.8% had cultural training. Most participants identified as Caucasian (75.4%) and were born in diverse countries. Perceptions of cultural competence were generally positive (M = 5.22/7), with lower scores in the "critical skills" domain. Age differences in perceptions were significant (p = 0.005).
Wasmuth 2020	USA	healthcare professionals	Community members and/or healthcare professionals	Likert-scale items created specifically for this study. Experiences of Discrimination18 (EOD)-report scale	Attitudes, Stereotype	Of 118 audience participants, 113 completed the post-show survey; 25% were healthcare providers. No significant differences were found in survey scores between providers and non-providers (Wilcoxon test, p = .302).

Table S7. PRISMA-ScR Checklist.

Preferred Reporting Items for Systematic reviews and Meta-Analyses extension for Scoping Reviews (PRISMA-ScR) Checklist

SECTION	ITEM	PRISMA-ScR CHECKLIST ITEM	REPORTED ON PAGE #
**TITLE**			
Title	1	Identify the report as a scoping review.	1
**ABSTRACT**			
Structured summary	2	Provide a structured summary that includes (as applicable): background, objectives, eligibility criteria, sources of evidence, charting methods, results, and conclusions that relate to the review questions and objectives.	1
**INTRODUCTION**			
Rationale	3	Describe the rationale for the review in the context of what is already known. Explain why the review questions/objectives lend themselves to a scoping review approach.	2-4
Objectives	4	Provide an explicit statement of the questions and objectives being addressed with reference to their key elements (e.g., population or participants, concepts, and context) or other relevant key elements used to conceptualize the review questions and/or objectives.	4
**METHODS**			
Protocol and registration	5	Indicate whether a review protocol exists; state if and where it can be accessed (e.g., a Web address); and if available, provide registration information, including the registration number.	4
Eligibility criteria	6	Specify characteristics of the sources of evidence used as eligibility criteria (e.g., years considered, language, and publication status), and provide a rationale.	4
Information sources*	7	Describe all information sources in the search (e.g., databases with dates of coverage and contact with authors to identify additional sources), as well as the date the most recent search was executed.	4

SECTION	ITEM	PRISMA-ScR CHECKLIST ITEM	REPORTED ON PAGE #
Search	8	Present the full electronic search strategy for at least 1 database, including any limits used, such that it could be repeated.	Supplementary File Table S1–S4
Selection of sources of evidence†	9	State the process for selecting sources of evidence (i.e., screening and eligibility) included in the scoping review.	4-6
Data charting process‡	10	Describe the methods of charting data from the included sources of evidence (e.g., calibrated forms or forms that have been tested by the team before their use, and whether data charting was done independently or in duplicate) and any processes for obtaining and confirming data from investigators.	4-7
Data items	11	List and define all variables for which data were sought and any assumptions and simplifications made.	4-7
Critical appraisal of individual sources of evidence§	12	If done, provide a rationale for conducting a critical appraisal of included sources of evidence; describe the methods used and how this information was used in any data synthesis (if appropriate).	Not applicable
Synthesis of results	13	Describe the methods of handling and summarizing the data that were charted.	7
**RESULTS**			
Selection of sources of evidence	14	Give numbers of sources of evidence screened, assessed for eligibility, and included in the review, with reasons for exclusions at each stage, ideally using a flow diagram.	6-7
Characteristics of sources of evidence	15	For each source of evidence, present characteristics for which data were charted and provide the citations.	7-8
Critical appraisal within sources of evidence	16	If done, present data on critical appraisal of included sources of evidence (see item 12).	Not applicable. Critical appraisal was not conducted.

SECTION	ITEM	PRISMA-ScR CHECKLIST ITEM	REPORTED ON PAGE #
Results of individual sources of evidence	17	For each included source of evidence, present the relevant data that were charted that relate to the review questions and objectives.	Supplementary Table S6
Synthesis of results	18	Summarize and/or present the charting results as they relate to the review questions and objectives.	8-9
**DISCUSSION**			
Summary of evidence	19	Summarize the main results (including an overview of concepts, themes, and types of evidence available), link to the review questions and objectives, and consider the relevance to key groups.	9-10
Limitations	20	Discuss the limitations of the scoping review process.	10
Conclusions	21	Provide a general interpretation of the results with respect to the review questions and objectives, as well as potential implications and/or next steps.	10
**FUNDING**			
Funding	22	Describe sources of funding for the included sources of evidence, as well as sources of funding for the scoping review. Describe the role of the funders of the scoping review.	11

JBI = Joanna Briggs Institute; PRISMA-ScR = Preferred Reporting Items for Systematic reviews and Meta-Analyses extension for Scoping Reviews.
* Where *sources of evidence* (see second footnote) are compiled from, such as bibliographic databases, social media platforms, and Web sites.
† A more inclusive/heterogeneous term used to account for the different types of evidence or data sources (e.g., quantitative and/or qualitative research, expert opinion, and policy documents) that may be eligible in a scoping review as opposed to only studies. This is not to be confused with *information sources* (see first footnote).
‡ The frameworks by Arksey and O’Malley (6) and Levac and colleagues (7) and the JBI guidance (4, 5) refer to the process of data extraction in a scoping review as data charting.
§ The process of systematically examining research evidence to assess its validity, results, and relevance before using it to inform a decision. This term is used for items 12 and 19 instead of "risk of bias" (which is more applicable to systematic reviews of interventions) to include and acknowledge the various sources of evidence that may be used in a scoping review (e.g., quantitative and/or qualitative research, expert opinion, and policy document).

From: Tricco AC, Lillie E, Zarin W, O'Brien KK, Colquhoun H, Levac D, et al. PRISMA Extension for Scoping Reviews (PRISMAScR): Checklist and Explanation. Ann Intern Med. 2018;169:467–473. doi: 10.7326/M18-0850.
